# Triplet Energy Transfer‐Driven Intermolecular [2+2] Photocycloaddition of Acyclic Imines and Indoles: Facile Access to Azetidine‐Fused Indolines

**DOI:** 10.1002/advs.202516247

**Published:** 2026-01-04

**Authors:** Gang Yang, Zeng Han, Hong Zhang, Xiuling Cui

**Affiliations:** ^1^ Key Laboratory of Fujian Molecular Medicine, Key Laboratory of Precision Medicine and Molecular Diagnosis of Fujian Universities Key Laboratory of Xiamen Marine and Gene Drugs, Engineering Research Centre of Molecular Medicine of Ministry of Education School of Biomedical Sciences Huaqiao University Xiamen P. R. China

**Keywords:** [2+2] photocycloaddition, aza Paternò‐Büchi reaction, azetidine‐fused indolines

## Abstract

Currently, successful examples of the aza‐[2+2] photocycloaddition (aza‐Paternò‐Büchi reaction) of indoles primarily rely on intramolecular variants or cyclic imine equivalents. To unlock the full synthetic potential of aza Paternò‐Büchi reaction in indoles, it is essential to extend the reaction to acyclic imine. Here, an intermolecular [2+2] photocycloaddition of indoles with acyclic imines has been developed, enabling visible light‐mediated aza‐Paternò‐Büchi reaction by triplet energy transfer. A range of azetidine‐fused indolines are successfully provided with yields of up to 93% for 30 examples. Given the growing significance of nitrogen‐containing heterocycles in pharmaceutical research and the challenge of their limited accessibility, this study afforded a novel synthetic methodology to construct previously inaccessible structural motifs with high molecular complexity by extending indole intermolecular [2+2] photocycloaddition with acyclic imines.

## Graphical abstract

An intermolecular aza‐Paternò‐Büchi reaction of indoles and acyclic imines is enabled by visible‐light‐driven triplet energy transfer, providing azetidine‐fused indolines up to 93% yield for 30 examples and providing a novel protocol to access previously inaccessible scaffolds.

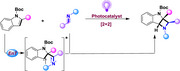

## 1 Introduction

Azetidine is a four‐membered saturated cyclic amine found in various natural products, which exhibits unique physiological functions, such as enhancing three‐dimensionality, improving pharmacokinetics, and reducing lipophilicity [1, 2, 3, 4, 5, 6, 7, 8, 9, 10]. And azetidine also possesses significant molecular rigidity (with a ring strain energy of 25.2 kcal/mol) and satisfactory stability [11]. Therefore, such a strained four‐membered ring containing nitrogen atom has successfully emerged as a privileged skeleton in the field of drug development and organic synthesis [12, 13]. However, azetidine‐containing pharmaceuticals remain markedly underrepresented among FDA‐approved drugs, comprising less than 1% of marketed medicines, in stark contrast to the widespread incorporation of five‐ and six‐membered heterocycles [14, 15, 16, 17, 18, 19, 20]. Despite the remarkable appeal of incorporating azetidine motif into complex molecular frameworks owing to their distinctive physicochemical characteristics, advancement has been hindered by the scarcity of efficient and general synthetic methodologies [21, 22]. Traditional strategies mainly involve intramolecular nucleophilic substitution of acyclic amines [23, 24, 25], reduction of lactam or *β*‐lactam [26, 27], and ring‐opening of strained azabicyclic intermediates [28, 29, 30, 31], yet remain limited in scope and efficiency. Their wider applicability is further limited by harsh reaction conditions, poor functional group tolerance, and the reliance on prefunctionalized substrates.

**SCHEME 1:**
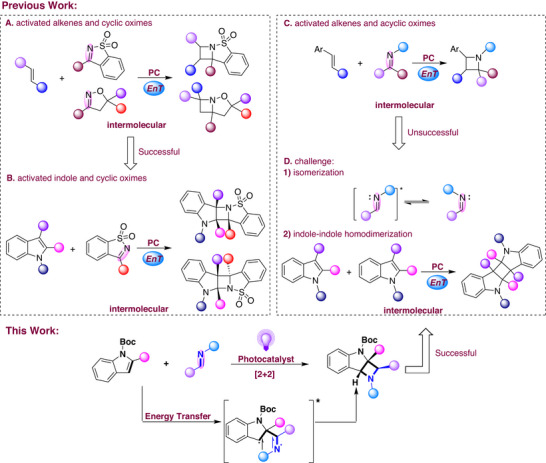
Previous Reports on the Construction of Azetidine and this Work.

Visible‐light‐mediated aza‐Paternò‐Büchi reaction has garnered vital attention as its being atom‐economical for the synthesis of highly functionalized azetidines [32, 33, 34, 35, 36, 37, 38, 39, 40, 41]. For example, Brown [42] and Schindler's groups [43] independently developed a visible‐light‐mediated intermolecular [2+2] cycloaddition of alkenes with 2‐isoxazoline‐3‐carboxylates, as well as alkenes with modulated *N*‐sulfonylimines, enabling the construction of a series of azetidines with high diastereo‐ and regioselectivity (Scheme 1A). Zhong's group demonstrated that triplet excited indoles, generated via sensitization, could promote diastereospecific [2+2] photocycloaddition with cyclic imines, resulting in the formation of polycyclic indolines (Scheme 1B) [44]. However, these procedures could be not applied for the acyclic imines, due to the challenges in capturing their excited state, which rapidly relaxes through isomerization. Schindler's group addressed such issue by matching the frontier orbital energies of the alkenes and the acyclic imines (Scheme 1C) [45, 46]. However, the reaction of indoles and acyclic imines remains unexplored. There are currently two challenges (Scheme 1D): (1) The imine chromophores exhibit low photoreactivity and undergo competing excited‐state processes such as *E*/*Z* isomerization, oxidation, and hydrolysis [47, 48, 49, 50]; (2) the formation of homodimers and heterodimers (H–H/H–T, endo/exo)occurs competitively [51, 52]. Based on that, we herein disclosed a visible‐light‐promoted TXT‐catalyzed intermolecular [2+2] cycloaddition of acyclic imines with indoles. The reaction proceeded smoothly via an energy transfer process under metal‐free and environmentally friendly conditions to afford azetidine‐fused indolines with excellent stereospecificity.

## 2 Results and Discussion

Initially, treating *tert*‐butyloxycarbonyl (Boc)‐protected indole‐2‐carboxyester **1a** with 2.0 equiv. of acyclic imine **2a** and 5 mol% of thioxanthen‐9‐one (TXT) in MeCN at 10 W LEDs for 20 h yielded the desired product **3aa** in 47% yield (Table 1, entry 1). The structure of **3aa** was confirmed through NMR, HRMS, and single‐crystal X‐ray diffraction analysis (see Supporting Information). Then the various parameters potentially affecting the efficiency of the formation of **3aa** were screened. The desired product **3aa** could not be afforded in darkness (Table 1, entry 2). Photocatalyst is crucial to this process, as no product could be detected in the absence of photocatalyst (Table 1, entry 3). The photocatalysts for the aza‐Paternò‐Büchi reaction most rely on Ir systems. While the inexpensive non‐metallic photocatalyst, such as thioxanthone (TXT), has not yet been successful for the [2+2] cycloaddition reaction of acyclic imines. Herein, screening photocatalyst shed light on that the TXT would be the best choice in the reaction. Other photosensitizer, such as 4CzIPN, [Ir(dF[CF_3_]ppy)_2_(dtbbpy)]PF_6_, Eosin Y, and Rhodamine B, exhibited lower efficiency compared to TXT, despite yielding positive results (Table 1, entries 4–7). Among the examined solvents, THF was optimal (Table 1, entry 8–13). Investigating the atmosphere (Table 1, entries 14–15) revealed that the yield of **3aa** in Air and O_2_ were 10% and 45%, respectively. Screening water scavengers revealed that 4Å molecular sieve (MS) was the most effective, providing a yield of 86%, whereas Na_2_SO_4_ and MgSO_4_ were less efficient (Table 1, entries 16–18). By examining the loading of **1a** and **2a**, it was found that the combination of **1a** (1.0 equiv.) and **2a** (1.2 equiv.) was optimal (Table 1, entries 19–22). Reducing the reaction time to 6 h decreased the yield from 86% to 63% (Table 1, entry 23). However, when the reaction time was decreased to 12 h and 16 h, the yield basically remained invariant (Table 1, entries 24–25). Furthermore, replacing the organic photocatalyst TXT with an [Ir(dF[CF_3_]ppy)_2_(dtbbpy)]PF_6_ under the optimized conditions resulted in a slightly lower yield of 74%, indicating that TXT is a more effective photocatalyst for this transformation (Table 1, entry 26).

**TABLE 1:** Reaction Condition Optimization.^a^

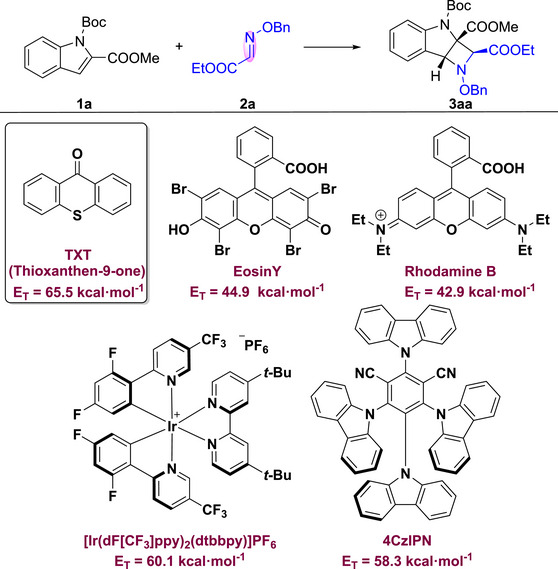					
Entry	PC	Solvent	Time (h)	1a: 2a	Yield (%)^b^
1	TXT	MeCN	20	1: 1.2	47
2^c^	Dark	MeCN	20	1: 1.2	0
3^d^	—	MeCN	20	1: 1.2	0
4	4CzIPN	MeCN	20	1: 1.2	40
5	[Ir(dF[CF_3_]ppy)_2_(dtbbpy)]PF_6_	MeCN	20	1: 1.2	45
6	Eosin Y	MeCN	20	1: 1.2	0
7	Rhodamine B	MeCN	20	1: 1.2	0
8	TXT	THF	20	1: 1.2	74
9	TXT	DCM	20	1: 1.2	70
10	TXT	HFIP	20	1: 1.2	0
11	TXT	TFE	20	1: 1.2	0
12	TXT	DMF	20	1: 1.2	0
13	TXT	DMSO	20	1: 1.2	0
14^e^	TXT	THF	20	1: 1.2	10
15^f^	TXT	THF	20	1: 1.2	45
16^g^	TXT	THF	20	1: 1.2	67
17^h^	TXT	THF	20	1: 1.2	64
18^i^	TXT	THF	20	1: 1.2	86
19^i^	TXT	THF	20	3: 1	86
20^i^	TXT	THF	20	1: 3	86
21^i^	TXT	THF	20	1: 1.0	85
22^i^	TXT	THF	20	1: 1.5	86
23^i^	TXT	THF	6	1: 1.2	63
24^i^	TXT	THF	12	1: 1.2	84
25^i^	TXT	THF	16	1: 1.2	85
26^i^	[Ir(dF[CF_3_]ppy)_2_(dtbbpy)]PF_6_	THF	12	1: 1.2	74

^a^ Conditions: **1a** (0.1 mmol), **2a** (0.12 mmol), PC (5 mol%), Solvent (2 mL), 10 W LEDs, room temperature, N_2_, 6–16 h.

^b^ Isolated Yield.

^c^ Dark.

^d^ Absence of the photocatalyst.

^e^ O_2_.

^f^ Air.

^g^ Na_2_SO_4_(0.2 mmol).

^h^ MgSO_4_ (0.2 mmol).

^i^ 4Å MS (20 mg).

**SCHEME 2:**
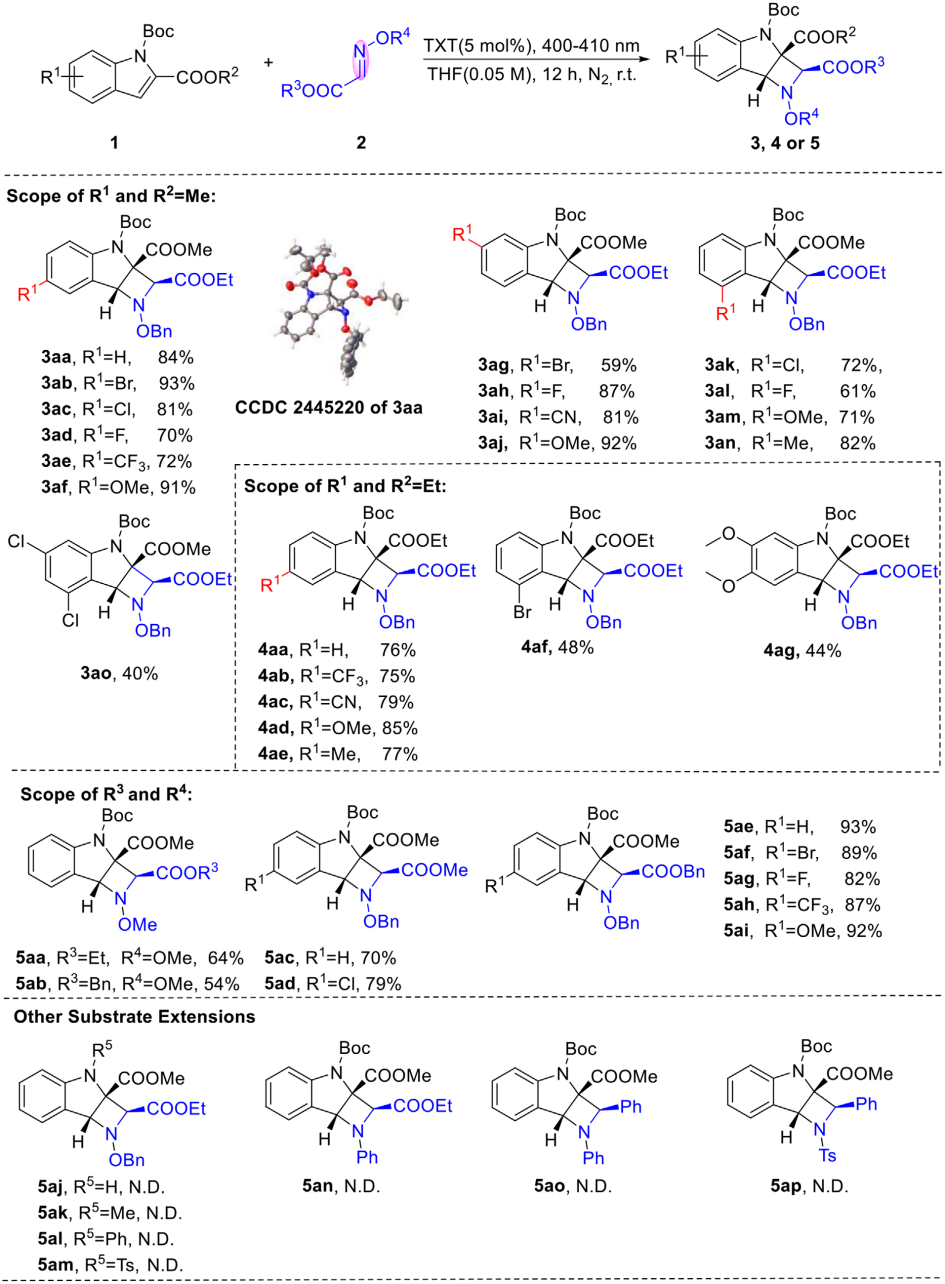
Scope of the Substrate. (a, b). (a) Reaction Conditions: **1** (0.1 mmol), **2** (0.12 mmol), TXT (5 mol%), 4Å MS (20 mg), 10 W LEDs (400–410 nm), THF (1.0 mL, N_2_, 12 h. (b) Isolated yields. N.D. = not detected.

With the optimized reaction conditions identified (Table 1, entry 24), we subsequently evaluated the generality of an array of indoles for this [2+2] cycloaddition reaction (Scheme 2). Various methyl formate substituted indoles (R^1^) could work well under the optimal conditions, giving fully substituted azetidines (**3aa**‐**3ao**) with good to excellent yields. Among which, the 4‐, 5‐, 6‐ or 7‐positions of the indole ring with the halogen atoms (F, Cl and Br) (**3ab**‐**3ad**, **3ag**‐**3ah**, **3ak**‐**3al**) were compatible in this reaction, delivering the corresponding azetidines in good to excellent yields (59%–93%), which provided potential opportunity for late‐stage functionalization. Both electron‐donating (‐Me, ‐OMe) and withdrawing (‐CN, ‐CF_3_) substituents at the 5‐ or 6‐positions of indole were well tolerated, affording the expected skeletons (**3ae**‐**3af**, **3ai**‐**3aj**) in 72%–92% yields. And electron‐donating (‐Me, ‐OMe) substituents at the 7‐position also could obtain the products **3am** and **3an** in 71% and 82% yields. These results indicated that the electronic property and steric hindrance slightly effected on the efficiency. Indoles substituted with dihalogen (Cl) could also furnish the target molecule **3ao** in 40% yield. When R^2^ was ethyl, the [2+2] cycloaddition reaction also proceeded well, furnishing the strained scaffold **4aa** in 76% yield. The desired products could be obtained in high yields and diastereoselectivity for both electron‐rich (**4ad**‐**4ae**) and electron‐deficient indoles (**4ab**‐**4ac**) in 75%‐85% yields, no significant impact of the electronic effect on the reaction was observed. At the 7‐position of indole with the Br atom provided product **4af** in the yield of 48%. Finally, disubstituted indole (‐OMe) also allowed for product construction (**4ag**), albeit in low yields (44%). The suitability of substrates containing variously substituted phenyl group at the acyclic imines (R^3^ and R^4^) was then evaluated (Scheme 2). Acyclic imines bearing either electron‐withdrawing or electron‐donating groups (**5aa**‐**5ai**) demonstrated good compatibility in the cycloaddition reaction. The benzyl ether group similarly yielded products (**5ac**‐**5ai**) in 70–93% yields. Various substituents at the 6‐position of the indole in the ‐OBn imines, including halogens (F and Br), electron‐donating (‐OMe), and electron‐withdrawing (‐CF_3_) groups, were well tolerated, giving the desired products (**5af**‐**5ai**) in excellent yields of 82%‐92%. However, the choice of imines ‐protecting group dramatically influenced the outcome, with ‐OMe (**5aa‐5ab**) imines providing products in lower yields, ranging from 54% to 64%. This result might be attributed to the stronger electron‐donating effect of ‐OMe compared to ‐OBn. While, attempts to replace the indole *N*‐protecting group (Boc) with *N*‐H, *N*‐Me, *N*‐Ph, or *N*‐Ts (**5aj**‐**5am**), as well as to employ other types of imines‐including *N*‐sulfonyl and *N*‐aryl imines (**5an**‐**5ap**) were unsuccessful.

**SCHEME 3:**
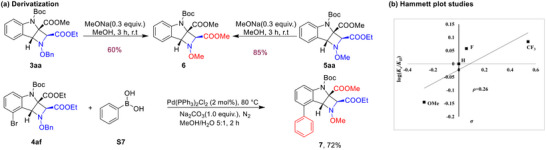
Derivatization and Hammett Plot Studies.

To enhance the synthetic utility of this protocol, post‐functionalization of the products was performed (Scheme 3a). For instance, products **3aa** and **5aa** underwent transesterification in methanol to afford products **6** in 60% and 85% yields, respectively. In addition, a palladium‐catalyzed Suzuki‐Miyaura cross‐coupling of **4af** with phenylboronic acid proceeded smoothly to give product **7** in 72% yield. Notably, as the reaction was carried out in methanol, the ester group was concomitantly converted from ‐COOEt to ‐COOMe. Hammett analysis was conducted using methyl *N*‐Boc‐indole‐2‐carboxylates (**1a**, **1d**, **1e**, and **1f**) (Table S1). Accordingly, the Hammett plot shows a moderate linear correlation, with a *ρ* value of 0.26, reflecting the electronic effect on the reaction rate (Scheme 3b).

**SCHEME 4:**
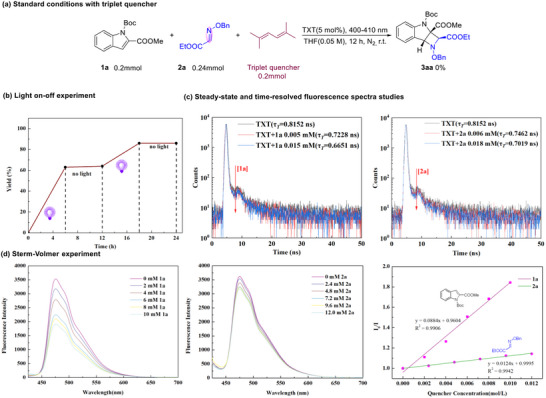
Standard conditions with triplet quencher (a); Light on‐off experiment (b); Steady‐state and time‐resolved fluorescence spectra studies (c); Sterm‐Volmer experiment(d).

To shed light on the reaction mechanism of the [2+2] cycloaddition reaction, experimental studies were conducted. In the presence of 1.0 equiv. of 2,5‐dimethylhexa‐2,4‐diene, a known triplet quencher, the standard reaction was significantly inhibited (Scheme 4a). Light on‐off experiments demonstrated that continuous visible‐light irradiation is essential (Scheme 4b). The oxidation and reduction potentials of **1a** (E_1/2_^ox^ = 1.97 V vs SCE, the corresponding reduction peak was not observed vs SCE) and **2a** (E_1/2_^red^ = ‐1.48 V vs SCE, the corresponding oxidation peak was not observed vs SCE) were measured by cyclic voltammetry. These potentials obtained fall out the redox range of TXT (E_1/2_^ox^ = 1.71 V vs SCE, E_1/2_^red^ = ‐1.03 V vs SCE); Thus, electron transfer between substrates **1a** and **2a** with TXT might not be involved in the reaction, which precluded a photoinduced redox process (Figure S3). The triplet lifetime of the photocatalyst TXT in THF was measured using steady‐state and time‐resolved emission spectroscopy. Upon addition of indole **1a** and imine **2a**, quenching the triplet state by **1a** was significantly more pronounced than that by **2a**. From the observed lifetime changes, the corresponding quenching rate constants (*k*_q_) were determined to be 1.75 × 10^13^ M^−1^·s^−1^ for **1a** and 1.04 × 10^13^ M^−1^·s^−1^ for **2a** (Table S2). These results demonstrate that triplet‐state quenching might be involved in this process (Scheme 4c). Furthermore, Stern‐Volmer quenching study revealed that the excited photocatalys was quenched by **1a**, whereas it was not detected for **2a** (Scheme 4d).

**SCHEME 5:**
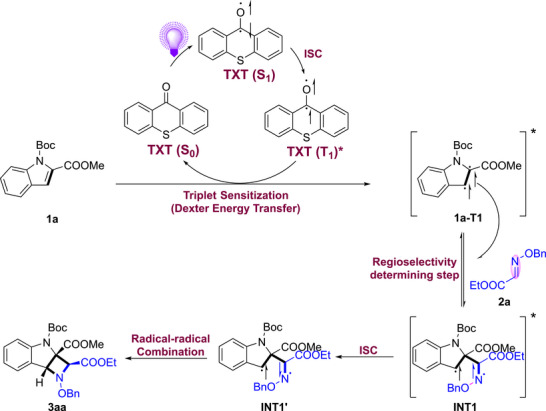
Plausible Mechanism.

Based on the above results and previous reports, [53, 54, 55, 56, 57, 58, 59, 60, 61] a photoinduced triplet‐energy‐transfer (EnT) pathway was proposed (Scheme 5). First, visible light was absorbed by the photocatalyst TXT, and its singlet state TXT(**S_1_**) was subsequently generated, after which rapid intersystem crossing (**ISC**) was undergone to give the triplet excited state TXT(**T_1_**). The reported triplet energy of TXT (65.5 kcal·mol^−1^) is significantly higher than that of indole **1a** (56.1 kcal·mol^−1^), indicating that the TXT→**1a** energy‐transfer process was thermodynamically favored. Through EnT, deactivation of TXT(**T_1_**) to TXT(**S_0_**) was accompanied by energy transfer to indole **1a**, and population of its triplet state was thus promoted, leading to formation of the reactive intermediate 1a* (**1a**‐**T_1_**). The C2‐centered radical of 1a* was then engaged in a regioselective addition to imine **2a**, and the triplet 1,4‐diradical intermediate **INT1** was formed. Finally, triplet‐to‐singlet spin inversion of **INT1** provided **INT1′**, which was then followed by radical–radical recombination, through which the cycloaddition product **3aa** was furnished.

## 3 Conclusion

In conclusion, the visible light‐mediated simultaneous activation of acyclic imines and indole for the construction of azetines via [2+2] annulation strategy has been demonstrated for the first time. The identification of *tert*‐butyloxycarbonyl (Boc)‐protected indole‐2‐carboxyesters as efficient substrates has enabled a dearomatizing intermolecular [2+2] cycloaddition with acyclic imines, effectively suppressing undesired homodimerization. This methodology afforded rapid, single‐step access to a broad spectrum of regio‐ and stereodefined azetidine‐fused indolines, serving as valuable scaffolds in pharmaceutical and agrochemical research. The visible‐light‐driven catalytic dearomatization provides a versatile platform for constructing 3D, sp^3^‐rich azetidine frameworks incorporating diverse functional groups from readily available precursors.

## Conflicts of Interest

The authors declare no conflicts of interest.

## Supporting information




**Supporting File**: advs73586‐sup‐0001‐SuppMat.docx.

## Data Availability

The data that support the findings of this study are available in the supplementary material of this article.
