# Supplementary material for: Triplet Energy Transfer‐Driven Intermolecular [2+2] Photocycloaddition of Acyclic Imines and Indoles: Facile Access to Azetidine‐Fused Indolines
Source: Adv Sci (Weinh). 2026 Jan 4;13(14):e16247. doi: 10.1002/advs.202516247 (PMC12970216; doi:10.1002/advs.202516247)
Supplement: Supplementary file 1 — Supporting File: advs73586‐sup‐0001‐SuppMat.docx. [file ADVS-13-e16247-s001.docx]

**Triplet Energy Transfer-Driven Intermolecular [2+2] Photocycloaddition of Acyclic Imines and Indoles: Facile Access to Azetidine-fused Indolines**

Gang Yang,^a^ Zeng Han,^a^ Hong Zhang^a^ and Xiuling Cui*^a^

^a^ Engineering Research Centre of Molecular Medicine of Ministry of Education, Key Laboratory of Fujian Molecular Medicine, Key Laboratory of Precision Medicine and Molecular Diagnosis of Fujian Universities, Key Laboratory of Xiamen Marine and Gene Drugs, School of Biomedical Sciences, Huaqiao University, Xiamen 361021, P. R. China; orcid.org/0000-0001-5759-766X; Email: cuixl@hqu.edu.cn.

**Content**

[**General information** 2](#_Toc215918471)

[**General procedure for synthesis of compound 1** 3](#_Toc215918472)

[**General procedure for synthesis of compound 2** 3](#_Toc215918473)

[**General procedure for synthesis of compound 3, 4 or 5** 4](#_Toc215918474)

[**General procedure for synthesis of compound 6** 5](#_Toc215918475)

[**General procedure for synthesis of compound 7** 5](#_Toc215918476)

[**Hammett Plot Analysis** 6](#_Toc215918477)

[**Light on-off experiment** 6](#_Toc215918478)

[**Stern-Volmer quenching studies** 7](#_Toc215918479)

[**Steady-state and time-resolved fluorescence spectra studies** 7](#_Toc215918480)

[**Cyclic voltammetry test** 7](#_Toc215918481)

[**Crystal data of compound 3aa** 8](#_Toc215918482)

[**Mechanistic investigations control experiment with triplet quenchers** 9](#_Toc215918483)

[**Characterization of the products** 10](#_Toc215918484)

[**Copies of NMR spectra** 25](#_Toc215918485)

[**References** 61](#_Toc215918486)

**General information**

All chemicals were obtained from commercial sources. The reactions were monitored by TLC. ^1^H NMR, ^13^C NMR and ^19^F NMR spectra were recorded on a Bruker 400 (400, 100 and 376 MHz) or Bruker 500 (500 and 126 MHz) spectrometer at room temperature in CDCl_3_ (solvent signals, *δ* 7.26 and 77.0 ppm) using TMS as internal standard. Mass spectra (MS) were measured on GCMS-QP2010 Ultra. High-resolution mass spectra (HRMS) were recorded on an electrospray ionization (ESI) apparatus using time-of-flight (TOF) mass spectrometry. Digital melting point apparatus was used to record the Melting Point of the compound in degree centigrade (°C) and are uncorrected. Electrochemical measurements were carried out on a CHI660D electrochemical workstation (CH Instruments, Shanghai, China) using a standard three-electrode system consisting of a glassy carbon working electrode, a platinum wire counter electrode, and an Hg/Hg_2_Cl_2_ reference electrode. Unless otherwise noted, all reactions were carried out using standard Schlenk techniques, and all starting materials and solvents were commercially available and used without further purification. Steady-state and time-resolved fluorescence spectra were recorded on a F1000 transient/steady-state fluorescence spectrometer (Edinburgh Instruments). Fluorescence spectra were recorded on an F98 spectrofluorometer (Shanghai Lengguang Technology Co., Ltd., China), previously known as Edinburgh Instruments. Column chromatography was performed on silica gel (300-400 mesh) using petroleum ether (PE)/ethyl acetate (EA). Thin-layer chromatography (TLC) was carried out on 4×5 cm plates with a layer thickness of 0.2 mm (silica gel 60 F254). Photochemical experiments were performed in a Parallel Light Reactor (designed by WATTCAS, WP-TEC-1020HSL, 10 W, *λ* = 400-410 nm, tube about 1~2 cm away from lights). The reaction setups were shown in **Figure S1**.


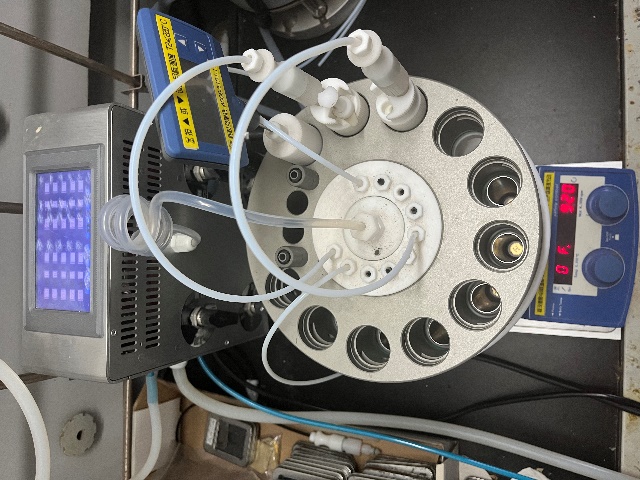


**Figure S1**. The WP-TEC-1020HSL photochemical reaction system.

**General procedure for synthesis of compound 1** ^[1]^

To an oven-dried 25 mL round-bottomed flask equipped with a magnetic stir bar were added indole-2-carboxylic acids **S1** (4.0 mmol, 1.0 equiv.), R^2^OH (10 mL), and H_2_SO_4_ (concentrated, 0.1 mL). The reaction was heated and refluxed for 24 h. After cooling to room temperature, water was added to the residue, the pH was adjusted to 7 by adding NaHCO_3_ (aq.), and the aqueous layer was extracted with EtOAc for three times. The solvent of combined organic layers was removed under reduced pressure and the resulting residue was purified by column chromatography on silica gel with petroleum ether/ethyl acetate (v/v) to afford the corresponding indole-2-carboxylates **S2**. Then, to a solution of indole-2-carboxylates **S2** (2.0 mmol) and DMAP (10 mol%) in MeCN (0.2 M), a solution of Boc_2_O (1.2 equiv.) in MeCN was added dropwise. The mixture was stirred for 24 hours at room temperature. Water was added to the residue, and the aqueous layer was extracted with EtOAc for three times. The solvent of combined organic layers was removed under reduced pressure and the resulting residue was purified by column chromatography on silica gel with petroleum ether/ethyl acetate (v/v) to afford the corresponding *N*-Boc-indole-2-carboxylates **1**.

**General procedure for synthesis of compound 2**

**Method 1**: Glyoxylic acid **S3** (1.0 equiv.) was dissolved in R^3^OH **S5**, o-benzylhydroxylamine hydrochloride (1.0 equiv.) **S4**. *p*-TsOH (0.1 equiv.) were then successively added. The mixture was heated at reflux for 5 hours. A Dean-Stark apparatus was then installed above the flask and ethanol was progressively distilled off. After an hour, most of the ethanol was removed and heating was stopped. The crude mixture was diluted with CH_2_Cl_2_. The organic phase was washed with saturated NaHCO_3_ and the resulting aqueous phase was back-extracted with CH_2_Cl_2_. The combined organic extracts were washed with saturated aqueous NaCl, dried over MgSO_4_, filtered and concentrated in vacuo. The residue was purified by column chromatography to give the products **2**. ^[2-3]^

**Method 2**: Acid **S3** (1.0 equiv.) was dissolved in water or THF (0.5-1.0 M), followed by addition of o-benzylhydroxylamine hydrochlorides **S4** (1.1 equiv.). Upon addition of most hydroxylamine hydrochloride salts, a thick white precipitate formed. If the solid hindered stirring, additional water was added to maintain stirring while keeping the mixture viscous. The reaction mixture was stirred overnight. Subsequently, the mixture was transferred to a separatory funnel containing water and diethyl ether. The aqueous layer was extracted five times with diethyl ether. The combined organic extracts were dried over MgSO_4_ and concentrated to afford the crude product as a white solid. This solid was recrystallized from a hexanes/ethyl acetate mixture by dissolving the crude product in hot hexanes and gradually adding ethyl acetate and warm hexanes until complete dissolution. The solution was cooled to room temperature, and placed in a -20 °C freezer for 2 h. The resulting crystals were collected by filtration and rinsed with cold hexanes to give products **S6**. Next, **S6** (1.1 or 1.2 equiv.) was dissolved in DMF (0.50 M) in a round-bottom flask. HATU (1.1 or 1.2 equiv.) was added, followed by **S5** (1.0 equiv.) and triethylamine (2.0 equiv.). The flask was sealed with a septum, purged with nitrogen, and stirred overnight. Upon completion, saturated ammonium chloride solution was added, and the reaction mixture was transferred to a separatory funnel with ethyl acetate. The aqueous layer was extracted three times with ethyl acetate. The combined organic layers were washed sequentially with 5% lithium chloride solution and brine, dried over Na_2_SO_4_, filtered, and concentrated. The crude product was purified by column chromatography using an ethyl acetate/hexanes eluent.^[4]^

**General procedure for synthesis of compound 3, 4 or 5**

To an over-dried quartz tube equipped with a magnetic stir bar was added with the mixture of methyl *N*-Boc-indole-2-carboxylate **1** (0.1 mmol), the acyclic imine **2** (0.12 mmol), thioxanthone (TXT, 1.1 mg, 5 mol%), 4Å MS (20 mg) in THF (2.0 mL). The reaction mixture was evacuated and backfilled with nitrogen three times, and then stirred under irradiation with violet LEDs (10 W, λ = 400-410 nm) for 12 h. After reaction completion, the solvent was removed under reduced pressure and the resulting residue was purified by column chromatography on silica gel with petroleum ether/ethyl acetate (v/v) to afford the corresponding azetidine-fused indolines **3**, **4** or **5**.

**General procedure for synthesis of compound 6**

To an oven-dried quartz tube equipped with a magnetic stir bar was added **3aa** or **5aa** (0.1 mmol) and MeONa (0.03 mmol, 0.3 equiv.) in MeOH (1.0 mL). The reaction mixture was stirred in air at room temperature for 3 h. Upon completion, the solvent was removed under reduced pressure, and the residue was purified by column chromatography on silica gel using petroleum ether/ethyl acetate (v/v = 10:1) as the eluent to afford the corresponding product **6**.

**General procedure for synthesis of compound 7**

To an oven-dried quartz tube equipped with a magnetic stir bar was charged with **4af** (0.1 mmol), phenylboronic acid **S7** (0.12 mmol), Pd(PPh_3_)_2_Cl_2_ (2 mol%), and Na_2_CO_3_ (0.1 mmol, 1.0 equiv.) under N_2_. MeOH/H_2_O (5:1, 1.0 mL) was added, and the reaction mixture was stirred at 80 °C for 2 h. Upon completion, the solvent was removed under reduced pressure, and the residue was purified by column chromatography on silica gel using petroleum ether/ethyl acetate (v/v = 15:1) as the eluent to afford the corresponding product 7.

**Hammett Plot Analysis**

To an over-dried quartz tube equipped with a magnetic stir bar was added with the mixture of methyl *N*-Boc-indole-2-carboxylates (**1a**, **1d**, **1e**, **1f**) (0.1 mmol), the acyclic imine **2a** (0.12 mmol), thioxanthone (TXT, 1.1 mg, 5 mol%), 4Å MS (20 mg) in THF (2.0 mL). The reaction mixture was evacuated and backfilled with nitrogen three times, and then stirred under irradiation with violet LEDs (10 W, λ = 400-410 nm) for 3 h. After reaction completion, the solvent was removed under reduced pressure and the resulting residue was purified by column chromatography on silica gel with petroleum ether/ethyl acetate (v/v) to afford the corresponding azetidine-fused indolines **3**.

Supplementary **Table 1**. Hammett plot studies

|  | *k*_x_ | *k*_x_/*k*_H_ | log(*k*_x_/*k*_H_) | *σ* |
| --- | --- | --- | --- | --- |
| OMe | 0.16 | 0.695652174 | 0.157607853 | -0.27 |
| H | 0.23 | 1 | 0 | 0 |
| F | 0.26 | 1.130434783 | 0.053245512 | 0.06 |
| CF_3_ | 0.27 | 1.173913043 | 0.069635928 | 0.54 |


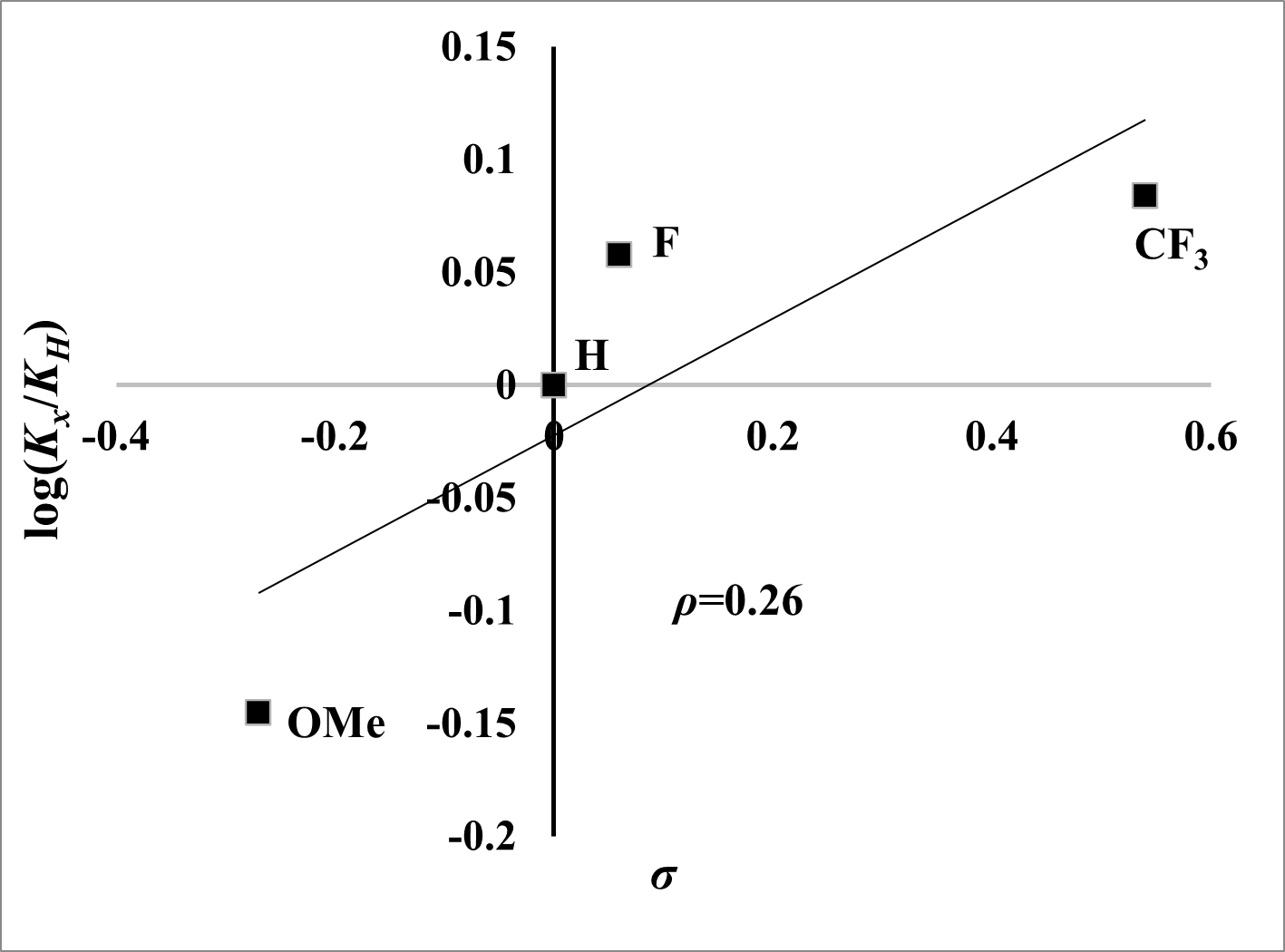


**Figure S2**. Hammett Plot Analysis

**Light on-off experiment**

To an over-dried quartz tube equipped with a magnetic stir bar was added with the mixture of methyl *N*-Boc-indole-2-carboxylate **1a** (0.1 mmol), the acyclic imine **2a** (0.12 mmol), thioxanthone (TXT, 1.1 mg, 5 mol%), 4Å MS (20 mg) in THF (2.0 mL). The reaction mixture was evacuated and backfilled with nitrogen three times. The reaction mixture was stirred in the dark or irradiated under violet LEDs (10 W, *λ* = 400-410 nm) for different periods. After reaction completion, the solvent was removed under the reduced pressure and the resulting residue was purified by column chromatography on silica gel with petroleum ether/ethyl acetate (v/v) to afford the product **3aa**.

**Stern-Volmer quenching studies**

Stern–Volmer quenching experiments were performed using a Lengguang F98 fluorescence spectrophotometer. All components were dissolved in acetonitrile immediately before use. The solutions were irradiated at 405 nm, and fluorescence emission was collected at 475 nm. Note: For practical reasons, [Ir(dF(CF_3_)ppy)_2_(dtbbpy)]PF_6_ was employed as the photocatalyst in place of thioxanthone (TXT). ^[5]^

**Steady-state and time-resolved fluorescence spectra studies**

Before each experiment, all component solutions were prepared in THF. The concentration of the photocatalyst TXT in all prepared solutions was maintained at 1 × 10^-5^ M.

Supplementary **Table 2**. Steady-state and time-resolved fluorescence spectra studies

| *τ_T_* | conc.( mM) (**1a**) | *τ*_0_/*τ* | *τ*_T_ | conc. (**2a**) | *τ*_0_/*τ* |
| --- | --- | --- | --- | --- | --- |
| 0.8152 | 0 mM | 1 | 0.8152 | 0 mM | 1 |
| 0.7228 | 0.005 mM | 1.127836 | 0.7462 | 0.006 mM | 1.092469 |
| 0.6651 | 0.015 mM | 1.22568 | 0.7019 | 0.018 mM | 1.161419 |
| *k*_q_(**1a**) | | 1.75×10^13^ M^-1^S^-1^ | *k*_q_(**2a**) | | 1.04×10^13^ M^-1^S^-1^ |

**Cyclic voltammetry test**

Cyclic voltammetry was conducted at ambient temperature in a conventional three-electrode setup, comprising a glassy carbon working electrode, a platinum wire counter electrode, and an Ag/AgCl reference electrode (saturated KCl). Measurements were performed in acetonitrile containing 0.1 M tetrabutylammonium hexafluorophosphate (*n*Bu_4_NPF_6_) as the supporting electrolyte, with a scan rate of 100 mV/s.

The CV data for TXT show that oxidation occurs at potential more positive than 1.71 V vs. Hg/Hg_2_Cl_2_ (sat. KCl), while reduction takes place at potential more negative than -1.03 V vs. Hg/Hg_2_Cl_2_ (sat. KCl). For **1a**, oxidation is observed at potential exceeding 1.97 V vs. Hg/ Hg_2_Cl_2_ (sat. KCl). For **2a**, reduction occurs at potential more negative than -1.48 V vs. Hg/ Hg_2_Cl_2_ (sat. KCl).

**Figure S3**. Cyclic voltammogram test of TXT, **1a** (0.01 M), **2a** (0.01 M) in MeCN (vs. Hg/Hg_2_Cl_2_, sat. KCl) with *n*Bu_4_NPF_6_ (0.1 M).

**Crystal data of compound 3aa**

Compound **3aa** (CCDC Number: 2445220)


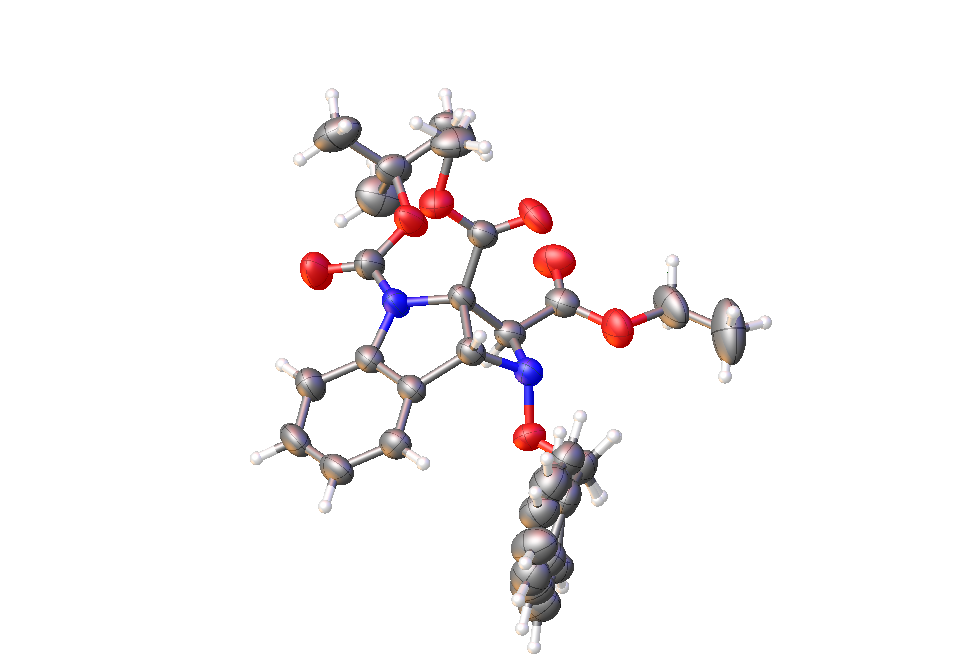


**Figure S4**. Crystal structure of **3aa** (CCDC Number: 2445220). Ellipsoids are drawn at the 50% probability level.

Crystallographic data for (**3aa**)

| Identification code | 2445220 |
| --- | --- |
| Empirical formula | C_26_H_30_N_2_O_7_ |
| Formula weight | 482.52 |
| Temperature/K | 298 |
| Crystal system | monoclinic |
| Space group | P21/c |
| a/Å | 9.9566(9) |
| b/Å | 23.805(2) |
| c/Å | 11.8185(12) |
| *α*/° | 90 |
| *β*/° | 113.597(3) |
| *γ*/° | 90 |
| Volume/Å3 | 2567.0(4) |
| Z | 4 |
| ρcalc g/cm3 | 1.249 |
| μ/mm‑1 | 0.091 |
| F(000) | 1024.0 |
| Crystal size/mm3 | 0.22 × 0.21 × 0.2 |
| Radiation | MoKα (*λ* = 0.71073) |
| 2ϴ range for data collection/° | 5.086 to 48.996 |
| Index ranges | -11 ≤ h ≤ 10, -27 ≤ k ≤ 27, 0 ≤ l ≤ 13 |
| Reflections collected | 8047 |
| Independent reflections | 4118 [Rint = 0.0893, Rsigma = 0.1174] |
| Data/restraints/parameters | 4118/159/376 |
| Goodness-of-fit on F2 | 1.108 |
| Final R indexes [I>=2σ (I)] | R1 = 0.0791, wR2 = 0.1918 |
| Final R indexes [all data] | R1 = 0.1100, wR2 = 0.2101 |
| Largest diff. peak/hole / e Å-3 | 0.31/-0.30 |

**Mechanistic investigations control experiment with triplet quenchers**

To an over-dried quartz tube equipped with a magnetic stir bar was added with the mixture of methyl *N*-Boc-indole-2-carboxylate **1a** (0.1 mmol), the acyclic imine (0.12 mmol), 2,5 dimethyl-2,4-hexadiene (0.1 mmol), thioxanthone (TXT, 1.1 mg, 5 mol%), 4Å MS (20 mg) in THF (2.0 mL). The reaction mixture was evacuated and backfilled with nitrogen three times, and then stirred under irradiation with violet LEDs (10 W, *λ* = 400-410 nm) for 12 h. After reaction completion, the solvent was removed under reduced pressure and the resulting residue was purified.

**Characterization of the products**

**3-(*tert*-butyl) 2-ethyl2a-methyl (2*S*,2a*S*,7b*R*)-1-(benzyloxy)-1,7b-dihydro-3*H*-azeto[3,2-*b*]indole-2,2a,3(2*H*)-tricarboxylate (3aa)**

Purified by flash column chromatography on silica gel (petroleum ether/EtOAc = 15:1 (v/v)). White solid. (40.5 mg, 84% yield). Mp = 117-118 °C. **^1^H NMR** (400 MHz, CDCl_3_) *δ* 7.93 (s, 1H), 7.32 (qd, *J* = 11.4, 4.6 Hz, 6H), 6.99 (d, *J* = 4.4 Hz, 2H), 5.32 (s, 1H), 4.95 (d, *J* = 11.5 Hz, 1H), 4.82 (d, *J* = 11.4 Hz, 1H), 4.44 – 4.32 (m, 2H), 4.32 – 4.18 (m, 1H), 3.75 (s, 3H), 1.49 (s, 9H), 1.35 (t, *J* = 7.1 Hz, 3H). **^13^C NMR** (100 MHz, CDCl_3_) *δ* 167.9, 167.5, 150.1, 144.5, 137.5, 130.3, 128.7, 128.3, 128.2, 127.9, 124.1, 123.0, 115.9, 82.7, 76.5, 76.3, 74.4, 66.3, 61.2, 52.7, 28.1, 14.3. HRMS (ESI-TOF) Calculated for C_26_H_30_N_2_NaO_7_^+^ ([M+Na^+^]): 505.1949. found: 505.1948.

**3-(*tert*-butyl) 2-ethyl 2a-methyl (2*S*,2a*S*,7b*R*)-1-(benzyloxy)-6-bromo-1,7b-dihydro-3*H*-azeo[3,2-*b*]indole-2,2a,3(2*H*)-tricarboxylate (3ab)**

Purified by flash column chromatography on silica gel (petroleum ether/EtOAc = 15:1 (v/v)). White solid. (52.1 mg, 93% yield). Mp = 117-118 °C.**^1^H NMR** (400 MHz, CDCl_3_) *δ* 7.81 (s, 1H), 7.44 – 7.36 (m, 5H), 7.36 – 7.29 (m, 2H), 6.94 (s, 1H), 5.25 (s, 1H), 4.95 (dd, *J* = 11.6, 1.6 Hz, 1H), 4.80 (dd, *J* = 11.5, 1.7 Hz, 1H), 4.44 – 4.36 (m, 1H), 4.34 (t, *J* = 1.4 Hz, 1H), 4.29 – 4.20 (m, 1H), 3.77 (s, 3H), 1.50 (s, 9H), 1.37 (t, *J* = 7.1 Hz, 3H). **^13^C NMR** (100 MHz, CDCl_3_) *δ* 167.7, 167.1, 150.0, 143.7, 137.5, 133.2, 131.2, 129.1, 128.6, 128.6, 128.3, 128.3, 126.3, 117.3, 115.5, 83.2, 76.7, 76.6, 74.0, 66.6, 61.4, 52.9, 28.1, 14.4. **HRMS** (ESI-TOF) Calculated for C_26_H_29_BrN_2_NaO_7_+ ([M+Na^+^]): 583.1050. found: 583.1050.

**3-(*tert*-butyl) 2-ethyl 2a-methyl (2*S*,2a*S*,7b*R*)-1-(benzyloxy)-6-chloro-1,7b-dihydro-3*H*-azeto[3,2-*b*]indole-2,2a,3(2*H*)-tricarboxylate (3ac)**

Purified by flash column chromatography on silica gel (petroleum ether/EtOAc = 15:1 (v/v)). White solid. (41.8 mg, 81% yield). Mp = 111-112 °C.**^1^H NMR** (400 MHz, CDCl_3_) *δ* 7.87 (s, 1H), 7.42 – 7.28 (m, 6H), 6.82 (s, 1H), 5.24 (s, 1H), 4.96 (d, *J* = 11.5 Hz, 1H), 4.81 (d, *J* = 11.5 Hz, 1H), 4.41 (dd, *J* = 18.0, 7.3 Hz, 1H), 4.34 (s, 1H), 4.31 – 4.21 (m, 1H), 3.78 (s, 3H), 1.50 (s, 9H), 1.37 (t, *J* = 8.4 Hz, 3H). **^13^C NMR** (100 MHz, CDCl_3_) *δ* 167.8, 167.3, 150.1, 143.3, 137.5, 130.4, 129.1, 128.7, 128.4, 128.3, 128.2, 125.9, 117.0, 83.3, 76.8, 76.7, 74.1, 66.7, 61.4, 53.0, 28.2, 14.4. **HRMS** (ESI-TOF) Calculated for C_26_H_30_ClN_2_O_7_^+^ ([M+H^+^]): 539.1556. found: 539.1556.

**3-(*tert*-butyl) 2-ethyl 2a-methyl (2*S*,2a*S*,7b*R*)-1-(benzyloxy)-6-fluoro-1,7b-dihydro-3*H*-azeto[3,2-*b*]indole-2,2a,3(2*H*)-tricarboxylate (3ad)**

Purified by flash column chromatography on silica gel (petroleum ether/EtOAc = 15:1 (v/v)). colorless oil. (35.1 mg, 70% yield). **^1^H NMR** (400 MHz, CDCl_3_) *δ* 7.89 (s, 1H), 7.41 – 7.35 (m, 3H), 7.35 – 7.28 (m, 2H), 7.02 (t, *J* = 8.9 Hz, 1H), 6.62 (d, *J* = 7.8 Hz, 1H), 5.25 (s, 1H), 4.96 (d, *J* = 11.5 Hz, 1H), 4.82 (d, *J* = 11.4 Hz, 1H), 4.43 – 4.21 (m, 3H), 3.78 (s, 3H), 1.50 (s, 9H), 1.41 – 1.33 (m, 3H). **^13^C NMR** (100 MHz, CDCl_3_) *δ* 167.9, 167.3, 158.8 (d, *J* = 242.4 Hz), 150.2, 140.6, 137.5, 129.1, 128.6, 128.3, 125.8 (d, *J* = 8.6 Hz), 117.0 (d, *J* = 23.1 Hz), 116.8 (d, *J* = 8.0 Hz), 115.3 (d, *J* = 24.4 Hz), 83.0, 76.7, 76.6, 74.1, 66.8, 61.4, 53.0, 28.2, 14.4. **^19^F NMR** (376 MHz, CDCl_3_) *δ* -120.15. **HRMS** (ESI-TOF) Calculated for C_26_H_29_FN_2_NaO_7_+ ([M+Na^+^]): 523.1851. found: 523.1850.

**3-(*tert*-butyl) 2-ethyl 2a-methyl (2*S*,2a*S*,7b*R*)-1-(benzyloxy)-6-(trifluoromethyl)-1,7b-dihydro-3*H*-azeto[3,2-*b*]indole-2,2a,3(2*H*)-tricarboxylate (3ae)**

Purified by flash column chromatography on silica gel (petroleum ether/EtOAc = 15:1 (v/v)). White solid. (39.7 mg, 72% yield). Mp = 149-150 °C.**^1^H NMR** (400 MHz, CDCl_3_) *δ* 8.01 (s, 1H), 7.58 (d, *J* = 8.7 Hz, 1H), 7.41 – 7.36 (m, 4H), 7.35 – 7.30 (m, 1H), 7.07 (s, 1H), 5.31 (s, 1H), 4.96 (d, *J* = 11.4 Hz, 1H), 4.80 (d, *J* = 13.0 Hz, 1H), 4.42 (d, *J* = 10.9 Hz, 1H), 4.35 (s, 1H), 4.33 – 4.23 (m, 1H), 3.79 (s, 3H), 1.52 (s, 9H), 1.39 (t, *J* = 7.2 Hz, 3H). **^13^C NMR** (100 MHz, CDCl_3_) *δ* 167.7, 167.1, 150.1, 147.6, 137.4, 129.2, 128.7, 128.4, 127.9 (q, J = 4.1 Hz), 125.7 (q, J = 26.0 Hz), 125.5, 125.1, 115.9, 83.7, 76.8, 76.8, 74.0, 66.9, 61.5, 53.0, 28.1, 14.4. **^19^F NMR** (376 MHz, CDCl_3_) *δ* -61.61. **HRMS** (ESI-TOF) Calculated for C_27_H_29_F_3_N_2_NaO_7_^+^ ([M+Na^+^]): 573.1819. found: 573.1819.

**3-(*tert*-butyl) 2-ethyl 2a-methyl (2*S*,2a*S*,7b*R*)-1-(benzyloxy)-6-methoxy-1,7b-dihydro-3*H*-azeto[3,2-*b*]indole-2,2a,3(2*H*)-tricarboxylate (3af)**

Purified by flash column chromatography on silica gel (petroleum ether/EtOAc = 15:1 (v/v)). colorless oil. (46.6mg, 91% yield). **^1^H NMR** (400 MHz, CDCl_3_) *δ* 7.84 (s, 1H), 7.40 (d, *J* = 7.3 Hz, 2H), 7.37 – 7.27 (m, 3H), 6.86 (d, *J* = 11.9 Hz, 1H), 6.48 (s, 1H), 5.28 (s, 1H), 4.98 (d, *J* = 14.3 Hz, 1H), 4.83 (d, *J* = 11.5 Hz, 1H), 4.40 (t, *J* = 7.5 Hz, 1H), 4.35 (s, 1H), 4.32 – 4.21 (m, 1H), 3.77 (s, 3H), 3.70 (s, 3H), 1.50 (s, 9H), 1.37 (t, *J* = 7.2 Hz, 3H). **^13^C NMR** (100 MHz, CDCl_3_) *δ* 168.1, 167.6, 155.8, 150.2, 138.0, 137.7, 129.0, 128.5, 128.1, 125.3, 116.6, 116.3, 113.4, 76.6, 76.4, 74.5, 66.6, 61.3, 55.7, 52.8, 28.2, 28.2, 14.4. **HRMS** (ESI-TOF) Calculated for C_27_H_32_N_2_NaO_8_^+^ ([M+Na^+^]): 535.2051. found: 535.2055.

**3-(*tert*-butyl) 2-ethyl 2a-methyl (2*S*,2a*S*,7b*R*)-1-(benzyloxy)-5-bromo-1,7b-dihydro-3*H*-azeto[3,2-*b*]indole-2,2a,3(2*H*)-tricarboxylate (3ag)**

Purified by flash column chromatography on silica gel (petroleum ether/EtOAc = 15:1 (v/v)). colorless oil. (33.1 mg, 59% yield). **^1^H NMR** (400 MHz, CDCl_3_) *δ* 8.17 (s, 1H), 7.39 – 7.30 (m, 5H), 7.14 (d, *J* = 9.2 Hz, 1H), 6.79 (d, *J* = 8.1 Hz, 1H), 5.25 (s, 1H), 4.95 (d, *J* = 11.4 Hz, 1H), 4.81 (d, *J* = 11.5 Hz, 1H), 4.40 (dd, *J* = 10.9, 7.1 Hz, 1H), 4.35 (s, 1H), 4.26 (dd, *J* = 11.0, 7.0 Hz, 1H), 3.78 (s, 3H), 1.51 (s, 9H), 1.37 (t, *J* = 7.2 Hz, 3H). **^13^C NMR** (100 MHz, CDCl_3_) *δ* 167.8, 167.2, 150.0, 145.8, 137.5, 129.3, 128.9, 128.5, 128.2, 126.2, 124.5, 123.3, 119.3, 83.4, 76.7, 76.6, 74.0, 66.8, 61.4, 53.0, 28.1, 14.4. **HRMS** (ESI-TOF) Calculated for C_26_H_29_BrN_2_NaO_7_^+^ ([M+Na^+^]): 583.1050. found: 583.1053.

**3-(*tert*-butyl) 2-ethyl 2a-methyl (2*S*,2a*S*,7b*R*)-1-(benzyloxy)-5-fluoro-1,7b-dihydro-3*H*-azeto[3,2-*b*]indole-2,2a,3(2*H*)-tricarboxylate (3ah)**

Purified by flash column chromatography on silica gel (petroleum ether/EtOAc = 15:1 (v/v)). colorless oil. (43.5 mg, 87% yield). **^1^H NMR** (400 MHz, CDCl_3_) *δ* 7.69 (s, 1H), 7.38 – 7.29 (m, 5H), 6.91 – 6.85 (m, 1H), 6.70 (t, *J* = 8.6 Hz, 1H), 5.27 (s, 1H), 4.95 (d, *J* = 11.3 Hz, 1H), 4.82 (d, *J* = 11.5 Hz, 1H), 4.45 – 4.33 (m, 2H), 4.32 – 4.19 (m, 1H), 3.78 (s, 3H), 1.51 (s, 9H), 1.37 (t, *J* = 7.1 Hz, 3H). **^13^C NMR** (100 MHz, CDCl_3_) *δ* 167.9, 167.3, 164.4 (d, *J* = 246.0 Hz), 150.0, 137.6, 129.1 (d, *J* = 10.4 Hz), 128.9, 128.5, 128.3, 128.2, 128.1, 119.7, 110.0 (d, *J* = 23.2 Hz), 104.2 (d, *J* = 29.6 Hz), 83.4, 76.6, 76.5, 73.8, 67.2, 61.5, 61.4, 52.9, 28.3, 28.1, 14.4. **^19^F NMR** (376 MHz, CDCl_3_) *δ* -109.99. **HRMS** (ESI-TOF) Calculated for C_26_H_29_FN_2_NaO_7_^+^ ([M+Na^+^]): 523.1851. found: 523.1850.

**3-(*tert*-butyl) 2-ethyl 2a-methyl (2*S*,2a*S*,7b*R*)-1-(benzyloxy)-5-cyano-1,7b-dihydro-3*H*-azeto[3,2-*b*]indole-2,2a,3(2*H*)-tricarboxylate (3ai)**

Purified by flash column chromatography on silica gel (petroleum ether/EtOAc = 15:1 (v/v)). colorless oil. (41.1 mg, 81% yield). **^1^H NMR** (400 MHz, CDCl_3_) *δ* 8.25 (s, 1H), 7.37 – 7.33 (m, 4H), 7.29 (s, 1H), 7.26 (d, *J* = 1.9 Hz, 1H), 6.96 (d, *J* = 7.8 Hz, 1H), 5.29 (s, 1H), 4.95 (d, *J* = 11.4 Hz, 1H), 4.80 (d, *J* = 11.5 Hz, 1H), 4.45 – 4.37 (m, 1H), 4.36 (s, 1H), 4.31 – 4.19 (m, 2H), 3.79 (s, 3H), 1.52 (s, 9H), 1.40 – 1.33 (m, 3H). **^13^C NMR** (100 MHz, CDCl_3_) *δ* 167.5, 166.9, 150.0, 145.3, 137.4, 129.1, 128.7, 128.4, 127.0, 119.2, 118.6, 114.2, 84.0, 77.4, 76.8, 74.1, 66.6, 61.6, 53.1, 28.1, 14.3. **HRMS** (ESI-TOF) Calculated for C_27_H_29_N_3_NaO_7_^+^ ([M+Na^+^]): 530.1898. found: 530.1899.

**3-(*tert*-butyl) 2-ethyl 2a-methyl (2*S*,2a*S*,7b*R*)-1-(benzyloxy)-5-methoxy-1,7b-dihydro-3*H*-azeto[3,2-*b*]indole-2,2a,3(2*H*)-tricarboxylate (3aj)**

Purified by flash column chromatography on silica gel (petroleum ether/EtOAc = 15:1 (v/v)). White solid. (47.1 mg, 92% yield). Mp = 168-169 °C.**^1^H NMR** (400 MHz, CDCl_3_) *δ* 7.58 (s, 1H), 7.41 – 7.27 (m, 5H), 6.88 (d, *J* = 8.4 Hz, 1H), 6.57 (d, *J* = 10.6 Hz, 1H), 5.28 (s, 1H), 4.95 (d, *J* = 13.4 Hz, 1H), 4.82 (d, *J* = 9.4 Hz, 1H), 4.47 – 4.32 (m, 2H), 4.31 – 4.19 (m, 1H), 3.79 (d, *J* = 18.7 Hz, 6H), 1.51 (s, 9H), 1.37 (t, *J* = 7.2 Hz, 3H). **^13^C NMR** (100 MHz, CDCl_3_) *δ* 168.1, 167.6, 161.8, 150.3, 137.7, 128.9, 128.7, 128.5, 128.1, 116.1, 110.1, 101.4, 82.9, 76.6, 76.4, 74.0, 67.2, 61.3, 55.7, 52.9, 28.2, 14.4. **HRMS** (ESI-TOF) Calculated for C_27_H_32_N_2_NaO_8_^+^ ([M+Na^+^]): 535.2051. found: 535.2051.

**3-(*tert*-butyl) 2-ethyl 2a-methyl (2*S*,2a*R*,7b*R*)-1-(benzyloxy)-7-chloro-7b-methyl-1,7b-dihydro-3*H*-azeto[3,2-*b*]indole-2,2a,3(2*H*)-tricarboxylate (3ak)**

Purified by flash column chromatography on silica gel (petroleum ether/EtOAc = 15:1 (v/v)). White solid. (38.2 mg, 72% yield). Mp = 112-113 °C.**^1^H NMR** (400 MHz, CDCl_3_) *δ* 7.92 – 7.86 (m, 1H), 7.40 – 7.23 (m, 4H), 7.08 (d, *J* = 8.0 Hz, 1H), 5.69 (s, 1H), 5.07 – 4.94 (m, 1H), 4.48 (s, 1H), 4.44 – 4.23 (m, 1H), 3.82 (s, 2H), 1.38 (t, *J* = 7.1 Hz, 2H). **^13^C NMR** (100 MHz, CDCl_3_) *δ* 167.6, 167.2, 150.1, 146.3, 136.8, 133.7, 132.1, 128.8, 128.6, 128.3, 128.0, 123.6, 123.3, 114.3, 83.4, 77.4, 77.2, 73.6, 66.3, 61.4, 53.0, 28.1, 14.4. **HRMS** (ESI-TOF) Calculated for C_26_H_29_ClN_2_NaO_7_^+^ ([M+Na^+^]): 539.1556. found: 539.1556.

**3-(*tert*-butyl) 2-ethyl 2a-methyl (2*S*,2a*R*,7b*R*)-1-(benzyloxy)-7-fluoro-7b-methyl-1,7b-dihydro-3*H*-azeto[3,2-*b*]indole-2,2a,3(2*H*)-tricarboxylate (3al)**

Purified by flash column chromatography on silica gel (petroleum ether/EtOAc = 15:1 (v/v)). White solid. (31.4 mg, 61% yield). Mp = 127-128 °C**.^1^H NMR** (400 MHz, CDCl_3_) *δ* 7.75 (s, 1H), 7.43 – 7.25 (m, 8H), 6.80 (t, *J* = 8.4 Hz, 1H), 5.68 (s, 1H), 5.01 – 4.88 (m, 2H), 4.42 (d, *J* = 8.0 Hz, 2H), 4.29 (dd, *J* = 10.9, 7.1 Hz, 1H), 3.82 (s, 3H), 1.54 (s, 9H), 1.39 (t, *J* = 7.1 Hz, 3H). **^13^C NMR** (100 MHz, CDCl_3_)*δ* 167.7, 167.2, 161.0 (d, *J* = 250.3 Hz), 150.1, 136.8, 132.8 (d, *J* = 8.3 Hz), 129.0, 128.4, 128.1, 111.9, 110.3 (d, *J* = 19.8 Hz), 83.4, 76.7, 76.6, 72.0, 66.8, 61.4, 53.1, 28.2, 14.4. **^19^F NMR** (376 MHz, CDCl_3_) *δ* -115.12. **HRMS** (ESI-TOF) Calculated for C_26_H_29_FN_2_NaO_7_^+^ ([M+Na^+^]): 523.1851. found: 523.1851.

**3-(*tert*-butyl) 2-ethyl 2a-methyl (2*S*,2a*S*,7b*R*)-1-(benzyloxy)-7-methoxy-1,7b-dihydro-3*H*-azeto[3,2-*b*]indole-2,2a,3(2*H*)-tricarboxylate (3am)**

Purified by flash column chromatography on silica gel (petroleum ether/EtOAc = 15:1 (v/v)). White solid. (36.4 mg, 71% yield). Mp = 143-144 °C. **^1^H NMR** (400 MHz, CDCl_3_) *δ* 7.59 (s, 1H), 7.40 – 7.23 (m, 6H), 6.61 (d, *J* = 8.2 Hz, 1H), 5.67 (s, 1H), 5.02 – 4.90 (m, 2H), 4.47 – 4.35 (m, 2H), 4.29 (dd, *J* = 10.9, 7.1 Hz, 1H), 3.79 (d, *J* = 11.5 Hz, 6H), 1.54 (s, 9H), 1.38 (t, *J* = 7.1 Hz, 3H). **^13^C NMR** (100 MHz, CDCl_3_) *δ* 168.0, 167.7, 158.3, 150.3, 137.5, 132.4, 128.3, 128.2, 127.7, 108.7, 105.6, 82.9, 76.6, 76.2, 72.8, 66.8, 61.3, 55.4, 52.9, 28.2, 14.4. **HRMS** (ESI-TOF) Calculated for C_27_H_32_N_2_NaO_8_^+^ ([M+Na^+^]): 535.2051. found: 535.2049.

**3-(*tert*-butyl) 2-ethyl 2a-methyl (2*S*,2a*S*,7b*R*)-1-(benzyloxy)-7-methyl-1,7b-dihydro-3*H*-azeto[3,2-*b*]indole-2,2a,3(2*H*)-tricarboxylate (3an)**

Purified by flash column chromatography on silica gel (petroleum ether/EtOAc = 15:1 (v/v)). White solid. (40.7 mg, 82% yield). Mp = 121-122 °C. **^1^H NMR** (400 MHz, CDCl_3_) *δ* 7.75 (s, 1H), 7.26 – 7.20 (m, 6H), 6.83 (d, *J* = 7.7 Hz, 1H), 5.55 (s, 1H), 4.99 – 4.84 (m, 2H), 4.44 (s, 1H), 4.42 – 4.28 (m, 1H), 4.21 (dq, *J* = 14.3, 7.2 Hz, 1H), 3.76 (s, 3H), 2.21 (s, 3H), 1.49 (s, 9H), 1.33 (t, *J* = 7.2 Hz, 3H). **^13^C NMR** (100 MHz, CDCl_3_) *δ* 168.1, 167.8, 138.2, 137.1, 130.8, 128.64, 128.4, 128.0, 124.6, 113.3, 82.8, 76.8, 74.1, 66.3, 61.4, 52.9, 28.2, 19.4, 14.4. **HRMS** (ESI-TOF) Calculated for C_27_H_32_N_2_NaO_7_^+^ ([M+Na^+^]): 519.2102 found: 519.2099.

**3-(*tert*-butyl) 2-ethyl 2a-methyl (2*S*,2a*S*,7b*R*)-1-(benzyloxy)-5,7-dichloro-1,7b-dihydro-3*H*-azeto[3,2-*b*]indole-2,2a,3(2*H*)-tricarboxylate (3ao)**

Purified by flash column chromatography on silica gel (petroleum ether/EtOAc = 15:1 (v/v)). White solid. (22 mg, 40% yield). Mp = 141-142 °C. **^1^H NMR** (400 MHz, CDCl_3_) *δ* 7.92 (s, 1H), 7.28 (s, 5H), 7.07 (d, *J* = 1.7 Hz, 1H), 5.60 (s, 1H), 5.00 – 4.90 (m, 2H), 4.43 (d, *J* = 1.6 Hz, 1H), 4.40 – 4.31 (m, 1H), 4.24 (dd, *J* = 10.8, 7.1 Hz, 1H), 3.79 (s, 4H), 3.60 (s, 1H), 1.51 (s, 9H), 1.35 (t, *J* = 7.1 Hz, 3H). **^13^C NMR** (100 MHz, CDCl_3_) *δ* 167.4, 166.8, 149.9, 146.9, 137.5, 136.7, 134.0, 128.8, 128.4, 128.1, 123.5, 122.0, 115.0, 84.0, 77.5, 77.2, 73.0, 66.7, 61.6, 53.1, 28.4, 28.1, 14.4. **HRMS** (ESI-TOF) Calculated for C_26_H_28_Cl_2_N_2_NaO_7_^+^ ([M+Na^+^]): 573.1166. found: 573.1169.

**3-(*tert*-butyl) 2,2a-diethyl (2*S*,2a*S*,7b*R*)-1-(benzyloxy)-1,7b-dihydro-3*H*-azeto[3,2-*b*]indole-2,2a,3(2*H*)-tricarboxylate (4aa)**

Purified by flash column chromatography on silica gel (petroleum ether/EtOAc = 15:1 (v/v)). White solid. (37.7 mg, 76% yield). Mp = 116-117 °C.**^1^H NMR** (400 MHz, CDCl_3_) *δ* 7.94 (s, 1H), 7.39 – 7.28 (m, 6H), 7.03 (s, 2H), 5.32 (s, 1H), 4.97 (d, *J* = 11.5 Hz, 1H), 4.84 (d, *J* = 13.2 Hz, 1H), 4.46 – 4.33 (m, 2H), 4.32 – 4.13 (m, 3H), 1.52 (s, 9H), 1.37 (t, *J* = 8.1 Hz, 3H), 1.26 (s, 3H). **^13^C NMR** (100 MHz, CDCl_3_) *δ* 168.2, 167.2, 150.4, 137.7, 130.4, 128.9, 128.5, 128.4, 128.1, 124.3, 123.1, 116.1, 82.9, 76.6, 76.5, 74.6, 66.5, 62.2, 61.3, 28.2, 14.4, 14.2. **HRMS** (ESI-TOF) Calculated for C_27_H_32_N_2_NaO_7_^+^ ([M+Na^+^]): 519.2102. found: 519.2101.

**3-(*tert*-butyl) 2,2a-diethyl (2*S*,2a*S*,7b*R*)-1-(benzyloxy)-6-(trifluoromethyl)-1,7b-dihydro-3*H*-azeto[3,2-*b*]indole-2,2a,3(2*H*)-tricarboxylate (4ab)**

Purified by flash column chromatography on silica gel (petroleum ether/EtOAc = 15:1 (v/v)). colorless oil. (42.3 mg, 75% yield). **^1^H NMR** (400 MHz, CDCl_3_) *δ* 8.04 (s, 1H), 7.61 (d, *J* = 8.7 Hz, 1H), 7.45 – 7.23 (m, 7H), 7.11 (s, 1H), 5.30 (s, 1H), 4.99 (d, *J* = 11.4 Hz, 1H), 4.83 (d, *J* = 11.4 Hz, 1H), 4.73 (d, *J* = 5.1 Hz, 1H), 4.43 (dt, *J* = 14.3, 7.1 Hz, 1H), 4.36 (s, 1H), 4.34 – 4.18 (m, 4H), 1.55 (s, 9H), 1.40 (t, *J* = 7.1 Hz, 3H), 1.30 (t, *J* = 7.0 Hz, 3H). **^13^C NMR** (100 MHz, CDCl_3_) *δ* 167.6, 166.6, 150.0, 137.3, 129.0, 128.6, 128.3, 128.3, 127.8, 125.6, 124.8, 115.8, 83.6, 77.2, 76.7, 76.6, 73.9, 66.8, 62.3, 61.3, 28.0, 14.3, 14.0. **^19^F NMR** (376 MHz, CDCl_3_) *δ* -61.68. **HRMS** (ESI-TOF) Calculated for C_28_H_31_F_3_N_2_NaO_7_^+^ ([M+Na^+^]): 587.1976. found: 587.1976.

**3-(*tert*-butyl) 2,2a-diethyl (2*S*,2a*S*,7b*R*)-1-(benzyloxy)-6-cyano-1,7b-dihydro-3*H*-azeto[3,2-*b*]indole-2,2a,3(2*H*)-tricarboxylate (4ac)**

Purified by flash column chromatography on silica gel (petroleum ether/EtOAc = 15:1 (v/v)). White solid. (41.2 mg, 79% yield). Mp = 103-104 °C. **^1^H NMR** (400 MHz, CDCl_3_) *δ* 8.00 (s, 1H), 7.60 (d, *J* = 11.4 Hz, 1H), 7.39 (d, *J* = 3.5 Hz, 4H), 6.98 (s, 1H), 5.21 (s, 1H), 4.95 (d, *J* = 11.5 Hz, 1H), 4.78 (d, *J* = 15.8 Hz, 1H), 4.47 – 4.36 (m, 1H), 4.33 (d, *J* = 2.8 Hz, 1H), 4.31 – 4.08 (m, 3H), 1.52 (s, 9H), 1.38 (d, *J* = 7.2 Hz, 3H), 1.30 – 1.24 (m, 3H). **^13^C NMR** (100 MHz, CDCl_3_) *δ* 167.5, 166.4, 149.9, 148.6, 137.4, 134.9, 132.1, 129.3, 128.8, 128.6, 128.3, 118.9, 116.4, 106.3, 84.2, 77.0, 73.7, 66.9, 62.5, 61.5, 28.1, 14.4, 14.1. **HRMS** (ESI-TOF) Calculated for C_28_H_31_N_3_NaO_7_^+^ ([M+Na^+^]): 544.2054. found: 544.2055.

**3-(*tert*-butyl) 2,2a-diethyl (2*S*,2a*S*,7b*R*)-1-(benzyloxy)-6-methoxy-1,7b-dihydro-3*H*-azeto[3,2-*b*]indole-2,2a,3(2*H*)-tricarboxylate (4ad)**

Purified by flash column chromatography on silica gel (petroleum ether/EtOAc = 15:1 (v/v)). colorless oil. (44.7 mg, 85% yield). **^1^H NMR** (400 MHz, CDCl_3_) *δ* 8.00 – 7.72 (m, 1H), 7.43 (d, *J* = 7.4 Hz, 2H), 7.40 – 7.31 (m, 3H), 6.89 (d, *J* = 8.9 Hz, 1H), 6.52 (s, 1H), 5.27 (s, 1H), 4.99 (d, *J* = 1.9 Hz, 1H), 4.90 – 4.83 (m, 1H), 4.42 (t, *J* = 5.8 Hz, 1H), 4.37 (d, *J* = 1.6 Hz, 1H), 4.33 – 4.15 (m, 3H), 3.73 (s, 3H), 1.53 (s, 9H), 1.39 (td, *J* = 7.1, 1.9 Hz, 3H), 1.32 – 1.27 (m, 3H). **^13^C NMR** (100 MHz, CDCl_3_) *δ* 168.1, 167.1, 155.7, 150.2, 138.0, 137.7, 128.9, 128.4, 128.0, 125.27, 116.6, 116.1, 113.3, 82.5, 76.5, 76.3, 74.4, 66.6, 62.1, 61.1, 28.1, 14.3, 14.0. **HRMS** (ESI-TOF) Calculated for C_28_H_34_N_2_NaO_8_^+^ ([M+Na^+^]): 549.2007. found: 549.2007.

**3-(*tert*-butyl) 2,2a-diethyl (2*S*,2a*S*,7b*R*)-1-(benzyloxy)-6-methyl-1,7b-dihydro-3*H*-azeto[3,2-*b*]indole-2,2a,3(2*H*)-tricarboxylate (4ae)**

Purified by flash column chromatography on silica gel (petroleum ether/EtOAc = 15:1 (v/v)). colorless oil. (39.3 mg, 77% yield). **^1^H NMR** (400 MHz, CDCl_3_) *δ* 7.81 (s, 1H), 7.39 – 7.34 (m, 3H), 7.35 – 7.30 (m, 2H), 7.13 (d, *J* = 8.4 Hz, 1H), 6.75 (s, 1H), 5.29 (s, 1H), 4.98 (d, *J* = 11.4 Hz, 1H), 4.84 (d, *J* = 11.5 Hz, 1H), 4.45 – 4.37 (m, 1H), 4.33 (s, 1H), 4.23 (dd, *J* = 25.7, 7.2 Hz, 3H), 2.28 (s, 3H), 1.51 (s, 9H), 1.37 (t, *J* = 7.1 Hz, 3H), 1.25 (d, *J* = 7.1 Hz, 3H). **^13^C NMR** (100 MHz, CDCl_3_) *δ* 168.2, 167.2, 150.4, 142.4, 137.8, 132.6, 131.0, 129.0, 128.9, 128.6, 128.5, 128.5, 128.1, 127.9, 124.3, 115.7, 82.7, 76.5, 76.5, 74.6, 66.6, 62.1, 61.2, 28.2, 20.9, 14.4, 14.4, 14.1. **HRMS** (ESI-TOF) Calculated for C_28_H_34_N_2_NaO_7_^+^ ([M+Na^+^]): 533.2258. found: 533.2256.

**3-(*tert*-butyl) 2,2a-diethyl (2*S*,2a*S*,7b*R*)-1-(benzyloxy)-7-bromo-1,7b-dihydro-3*H*-azeto[3,2-*b*]indole-2,2a,3(2*H*)-tricarboxylate (4af)**

Purified by flash column chromatography on silica gel (petroleum ether/EtOAc = 15:1 (v/v)). White solid. (27.6 mg, 48% yield). Mp = 104-105 °C. **^1^H NMR** (400 MHz, CDCl_3_) *δ* 7.92 (s, 1H), 7.34 – 7.24 (m, 5H), 7.22 (d, *J* = 4.5 Hz, 2H), 5.60 (s, 1H), 5.06 (d, *J* = 10.4 Hz, 1H), 4.97 (d, *J* = 10.5 Hz, 1H), 4.46 (d, *J* = 1.6 Hz, 1H), 4.37 (dd, *J* = 10.9, 7.1 Hz, 1H), 4.34 – 4.14 (m, 4H), 1.52 (s, 9H), 1.35 (t, *J* = 7.1 Hz, 3H), 1.29 (t, *J* = 7.2 Hz, 3H). **^13^C NMR** (100 MHz, CDCl_3_) *δ* 167.6, 166.7, 150.1, 146.2, 136.9, 132.1, 128.7, 128.6, 128.3, 128.3, 127.9, 126.4, 125.49, 122.49, 114.77, 83.35, 77.5, 77.2, 74.8, 66.0, 62.4, 61.3, 28.1, 14.3, 14.2, 14.1. **HRMS** (ESI-TOF) Calculated for C_27_H_31_BrN_2_NaO_7_^+^ ([M+Na^+^]): 597.1207. found: 597.1207.

**3-(*tert*-butyl) 2,2a-diethyl (2*S*,2a*S*,7b*R*)-1-(benzyloxy)-5,6-dimethoxy-1,7b-dihydro-3*H*-azeto[3,2-*b*]indole-2,2a,3(2*H*)-tricarboxylate (4ag)**

Purified by flash column chromatography on silica gel (petroleum ether/EtOAc = 15:1 (v/v)). colorless oil. (24.5 mg, 44% yield). **^1^H NMR** (400 MHz, CDCl_3_) *δ* 7.66 (d, *J* = 6.3 Hz, 1H), 7.40 (dd, *J* = 8.0, 1.9 Hz, 2H), 7.37 – 7.27 (m, 3H), 7.26 (s, 1H), 6.39 (s, 1H), 5.26 (s, 1H), 4.99 (d, *J* = 11.4 Hz, 1H), 4.83 (d, *J* = 11.5 Hz, 1H), 4.44 – 4.35 (m, 1H), 4.33 (s, 1H), 4.31 – 4.22 (m, 2H), 4.22 – 4.11 (m, 2H), 3.95 (s, 1H), 3.91 (d, *J* = 3.1 Hz, 3H), 3.73 (s, 3H), 1.51 (s, 9H), 1.40 – 1.34 (m, 4H), 1.27 (t, *J* = 7.2 Hz, 5H). **^13^C NMR** (100 MHz, CDCl_3_) *δ* 168.2, 167.2, 150.7, 150.4, 145.2, 137.9, 129.0, 128.5, 128.2, 110.7, 100.3, 82.7, 76.6, 76.4, 67.0, 62.2, 61.5, 61.3, 56.3, 56.2, 28.2, 28.1, 14.4, 14.3, 14.2. **HRMS** (ESI-TOF) Calculated for C_29_H_36_N_2_NaO_9_^+^ ([M+Na^+^]): 579.2313. found: 579.2312.

**3-(*tert*-butyl) 2-ethyl 2a-methyl (2*S*,2a*S*,7b*R*)-1-methoxy-1,7b-dihydro-3*H*-azeto[3,2-*b*]indole-2,2a,3(2*H*)-tricarboxylate (5aa)**

Purified by flash column chromatography on silica gel (petroleum ether/EtOAc = 15:1 (v/v)). White solid. (26.0 mg, 64% yield). Mp = 128-129 °C. **^1^H NMR** (400 MHz, CDCl_3_) *δ* 7.97 (s, 1H), 7.36 (t, *J* = 7.9 Hz, 1H), 7.29 (d, *J* = 7.6 Hz, 1H), 7.08 (t, *J* = 7.5 Hz, 1H), 5.49 (s, 1H), 4.39 (ddd, *J* = 15.2, 10.7, 6.9 Hz, 1H), 4.34 – 4.18 (m, 2H), 3.78 (s, 3H), 3.66 (s, 4H), 1.51 (s, 9H), 1.36 (t, *J* = 7.2 Hz, 3H). **^13^C NMR** (100 MHz, CDCl_3_) *δ* 168.0, 167.5, 150.2, 144.4, 130.4, 128.2, 124.1, 123.1, 116.0, 82.8, 77.1, 76.3, 73.7, 66.0, 61.5, 61.3, 52.8, 28.1, 14.3. **HRMS** (ESI-TOF) Calculated for C_20_H_26_N_2_NaO_7_^+^ ([M+Na^+^]):429.1632. found: 529.1630.

**2-benzyl 3-(*tert*-butyl) 2a-methyl (2*S*,2a*S*,7b*R*)-1-methoxy-1,7b-dihydro-3*H*-azeto[3,2-*b*]indole-2,2a,3(2*H*)-tricarboxylate (5ab)**

Purified by flash column chromatography on silica gel (petroleum ether/EtOAc = 15:1 (v/v)). White solid. (25.3 mg, 54% yield). Mp = 103-104 °C. **^1^H NMR** (400 MHz, CDCl_3_) *δ* 7.93 (s, 1H), 7.40 (d, *J* = 7.3 Hz, 2H), 7.31 (dd, *J* = 17.5, 7.3 Hz, 5H), 7.06 (t, *J* = 7.5 Hz, 1H), 5.49 (s, 1H), 5.39 (d, *J* = 12.4 Hz, 1H), 5.20 (d, *J* = 12.3 Hz, 1H), 4.33 (d, *J* = 2.1 Hz, 1H), 3.71 (d, *J* = 2.0 Hz, 3H), 3.62 (d, *J* = 1.9 Hz, 3H), 1.39 (s, 9H). **^13^C NMR** (100 MHz, CDCl_3_) *δ* 168.0, 167.5, 150.2, 144.4, 135.7, 130.5, 128.5, 128.5, 128.3, 128.3, 124.1, 123.2, 116.1, 82.9, 76.2, 73.8, 67.0, 66.1, 61.5, 52.9, 28.0. **HRMS** (ESI-TOF) Calculated for C_25_H_28_N_2_NaO_7_^+^ ([M+Na^+^]): 491.1789. found: 491.1791.

**3-(*tert*-butyl) 2,2a-dimethyl (2*S*,2a*S*,7b*R*)-1-(benzyloxy)-1,7b-dihydro-3*H*-azeto[3,2-*b*]indole-2,2a,3(2*H*)-tricarboxylate (5ac)**

Purified by flash column chromatography on silica gel (petroleum ether/EtOAc = 15:1 (v/v)). White solid. (39.8 mg, 79% yield). Mp = 130-131 °C.**^1^H NMR** (400 MHz, CDCl_3_) *δ* 8.03 – 7.89 (m, 1H), 7.40 – 7.27 (m, 7H), 7.02 (d, *J* = 6.0 Hz, 2H), 5.35 (s, 1H), 4.97 (dd, *J* = 11.5, 2.0 Hz, 1H), 4.83 (dd, *J* = 11.3, 2.1 Hz, 1H), 4.41 – 4.37 (m, 1H), 3.83 (d, *J* = 2.0 Hz, 3H), 3.77 (d, *J* = 2.0 Hz, 3H), 1.51 (s, 9H). **^13^C NMR** (100 MHz, CDCl_3_) *δ* 168.6, 167.6, 150.1, 144.5, 137.5, 130.4, 128.8, 128.4, 128.3, 128.1, 128.0, 124.0, 123.1, 115.9, 82.8, 77.1, 76.4, 76.4, 74.5, 66.3, 52.8, 52.1, 28.1. **HRMS** (ESI-TOF) Calculated for C_25_H_28_N_2_NaO_7_^+^ ([M+Na^+^]): 491.1789. found:491.1789.

**3-(*tert*-butyl) 2,2a-dimethyl (2*S*,2a*S*,7b*R*)-1-(benzyloxy)-6-chloro-1,7b-dihydro-3*H*-azeto[3,2-*b*]indole-2,2a,3(2*H*)-tricarboxylate (5ad)**

Purified by flash column chromatography on silica gel (petroleum ether/EtOAc = 15:1 (v/v)). White solid. (39.7 mg, 79% yield). Mp = 109-110 °C. **^1^H NMR** (400 MHz, CDCl_3_) *δ* 7.88 (d, *J* = 9.1 Hz, 1H), 7.42 – 7.36 (m, 4H), 7.36 – 7.28 (m, 2H), 7.28 – 7.25 (m, 1H), 6.80 (d, *J* = 2.2 Hz, 1H), 5.25 (s, 1H), 4.96 (d, *J* = 11.5 Hz, 1H), 4.81 (d, *J* = 11.5 Hz, 1H), 4.36 (d, *J* = 1.5 Hz, 1H), 3.84 (s, 3H), 3.78 (s, 3H), 1.49 (s, 9H). **^13^C NMR** (100 MHz, CDCl_3_) *δ* 168.3, 167.3, 150.0, 143.3, 137.5, 130.4, 129.1, 128.6, 128.4, 128.3, 128.3, 125.8, 116.9, 83.2, 76.8, 76.5, 74.1, 66.7, 53.0, 52.3, 28.1. **HRMS** (ESI-TOF) Calculated for C_25_H_27_ClN_2_NaO_7_^+^ ([M+Na^+^]): 525.1399. found: 525.1398.

**2-benzyl 3-(*tert*-butyl) 2a-methyl (2*S*,2a*S*,7b*R*)-1-(benzyloxy)-1,7b-dihydro-3*H*-azeto[3,2-*b*]indole-2,2a,3(2*H*)-tricarboxylate (5ae)**

Purified by flash column chromatography on silica gel (petroleum ether/EtOAc = 15:1 (v/v)). White solid. (50.6 mg, 93% yield). Mp = 110-111 °C. **^1^H NMR** (400 MHz, CDCl_3_) *δ* 7.93 (s, 1H), 7.44 (d, *J* = 7.2 Hz, 2H), 7.38 – 7.29 (m, 9H), 7.02 (d, *J* = 4.3 Hz, 2H), 5.43 (d, *J* = 12.5 Hz, 1H), 5.34 (s, 1H), 5.29 (s, 1H), 5.24 (s, 1H), 4.96 (s, 1H), 4.80 (d, *J* = 11.4 Hz, 1H), 4.45 (s, 1H), 3.74 (s, 3H), 1.43 (s, 9H). **^13^C NMR** (100 MHz, CDCl_3_) *δ* 167.9, 167.5, 150.2, 144.4, 137.5, 135.8, 130.4, 128.9, 128.5, 128.5, 128.4, 128.3, 128.0, 124.1, 123.1, 115.9, 76.5, 76.4, 74.5, 66.9, 66.3, 52.8, 28.0. **HRMS** (ESI-TOF) Calculated for C_31_H_32_N_2_NaO_7_^+^ ([M+Na^+^]): 567.2102. found: 567.2103.

**2-benzyl 3-(*tert*-butyl) 2a-methyl (2*S*,2a*S*,7b*R*)-1-(benzyloxy)-6-bromo-1,7b-dihydro-3*H*-azeto[3,2-*b*]indole-2,2a,3(2*H*)-tricarboxylate (5af)**

Purified by flash column chromatography on silica gel (petroleum ether/EtOAc = 15:1 (v/v)). colorless oil. (55.4 mg, 89% yield). **^1^H NMR** (400 MHz, CDCl_3_) *δ* 7.79 (s, 1H), 7.43 (d, *J* = 7.1 Hz, 3H), 7.36 (d, *J* = 8.0 Hz, 8H), 6.94 (s, 1H), 5.42 (d, *J* = 12.2 Hz, 1H), 5.22 (d, *J* = 15.8 Hz, 2H), 4.91 (d, *J* = 9.5 Hz, 1H), 4.76 (d, *J* = 11.5 Hz, 1H), 4.40 (s, 1H), 3.72 (s, 3H), 1.40 (s, 9H). **^13^C NMR** (100 MHz, CDCl_3_) *δ* 167.7, 167.2, 150.1, 137.5, 135.8, 133.3, 131.3, 129.2, 128.7, 128.6, 128.6, 128.5, 128.3, 117.4, 115.6, 83.40, 76.7, 76.6, 67.1, 66.7, 53.0, 28.1. **HRMS** (ESI-TOF) Calculated for C_31_H_31_BrN_2_NaO_7_^+^ ([M+Na^+^]): 645.1207. found: 645.1207.

**2-benzyl 3-(*tert*-butyl) 2a-methyl (2*S*,2a*S*,7b*R*)-1-(benzyloxy)-6-fluoro-1,7b-dihydro-3*H*-azeto[3,2-*b*]indole-2,2a,3(2*H*)-tricarboxylate (5ag)**

Purified by flash column chromatography on silica gel (petroleum ether/EtOAc = 15:1 (v/v)). colorless oil. (46.1 mg, 82% yield). **^1^H NMR** (400 MHz, CDCl_3_) *δ* 7.88 (s, 1H), 7.44 (d, *J* = 7.7 Hz, 2H), 7.34 (q, *J* = 6.5 Hz, 8H), 7.02 (t, *J* = 8.9 Hz, 1H), 6.62 (d, *J* = 7.8 Hz, 1H), 5.42 (d, *J* = 12.3 Hz, 1H), 5.22 (d, *J* = 15.3 Hz, 2H), 4.92 (d, *J* = 11.4 Hz, 1H), 4.77 (d, *J* = 11.4 Hz, 1H), 4.43 (s, 1H), 3.74 (s, 3H), 1.41 (s, 9H). **^13^C NMR** (100 MHz, CDCl_3_) *δ* 167.9, 167.3, 158.8 (d, *J* = 242.4 Hz), 150.2, 140.6, 137.5, 135.8, 129.1, 128.8, 128.8, 128.6, 128.6, 128.4, 128.29, 128.2, 117.1 (d, *J* = 23.1 Hz), 116.8 (d, *J* = 8.0 Hz), 115.4 (d, *J* = 24.3 Hz), 83.2, 76.7, 76.6, 74.1, 67.1, 66.8, 53.0, 28.1. **^19^F NMR** (376 MHz, CDCl_3_) *δ* -120.14. **HRMS** (ESI-TOF) Calculated for C_31_H_31_FN_2_NaO_7_^+^ ([M+Na^+^]): 585.2008. found: 585.2009.

**2-benzyl 3-(*tert*-butyl) 2a-methyl (2*S*,2a*S*,7b*R*)-1-(benzyloxy)-6-(trifluoromethyl)-1,7b-dihydro-3*H*-azeto[3,2-*b*]indole-2,2a,3(2*H*)-tricarboxylate (5ah)**

Purified by flash column chromatography on silica gel (petroleum ether/EtOAc = 15:1 (v/v)). colorless oil. (53.3 mg, 87% yield). **^1^H NMR** (400 MHz, CDCl_3_) *δ* 8.00 (s, 1H), 7.58 (d, *J* = 8.7 Hz, 1H), 7.44 (d, *J* = 7.4 Hz, 2H), 7.35 (q, *J* = 8.0 Hz, 8H), 7.06 (s, 1H), 5.42 (d, *J* = 12.3 Hz, 1H), 5.29 (s, 1H), 5.23 (d, *J* = 12.2 Hz, 1H), 4.92 (d, *J* = 11.4 Hz, 1H), 4.75 (d, *J* = 11.4 Hz, 1H), 4.41 (s, 1H), 3.73 (s, 3H), 1.42 (s, 9H). **^13^C NMR** (100 MHz, CDCl_3_) *δ* 167.5, 167.0, 149.9, 147.4, 137.2, 135.6, 129.1, 128.6, 128.5, 128.5, 128.4, 128.3, 127.8 (q, *J* = 3.3 Hz), 125.6, 125.4, 125.3, 125.0, 124.7, 122.7, 115.8, 83.7, 77.2, 76.6, 73.9, 67.1, 66.8, 52.9, 27.9. **^19^F NMR** (376 MHz, CDCl_3_) *δ* -61.70. **HRMS** (ESI-TOF) Calculated for C_32_H_31_F_3_N_2_NaO_7_^+^ ([M+Na^+^]): 635.1976. found: 635.1978.

**2-benzyl 3-(*tert*-butyl) 2a-methyl (2*S*,2a*S*,7b*R*)-1-(benzyloxy)-6-methoxy-1,7b-dihydro-3H-azeto[3,2-*b*]indole-2,2a,3(2*H*)-tricarboxylate (5ai)**

Purified by flash column chromatography on silica gel (petroleum ether/EtOAc = 15:1 (v/v)). colorless oil. (52.8 mg, 92% yield). **^1^H NMR** (400 MHz, CDCl_3_) *δ* 7.82 (s, 1H), 7.44 (d, *J* = 7.4 Hz, 2H), 7.34 (h, *J* = 7.3 Hz, 8H), 6.87 (d, *J* = 13.8 Hz, 1H), 6.47 (s, 1H), 5.43 (d, *J* = 12.3 Hz, 1H), 5.29 – 5.16 (m, 2H), 4.95 (d, *J* = 11.1 Hz, 1H), 4.79 (d, *J* = 11.4 Hz, 1H), 4.42 (s, 1H), 3.74 (s, 3H), 3.71 (s, 3H), 1.40 (s, 9H). **^13^C NMR** (100 MHz, CDCl_3_) *δ* 168.1, 167.6, 155.8, 150.3, 137.7, 135.9, 129.1, 128.7, 128.6, 128.5, 128.4, 128.2, 116.7, 116.3, 113.4, 82.7, 76.7, 76.4, 74.6, 67.0, 66.7, 55.8, 52.9, 28.1. **HRMS** (ESI-TOF) Calculated for C_32_H_34_N_2_NaO_8_^+^ ([M+Na^+^]): 597.2207. found: 597.2204.

**3-(*tert*-butyl) 2,2a-dimethyl (2*S*,2a*S*,7b*R*)-1-methoxy-1,7b-dihydro-3*H*-azeto[3,2-*b*]indole-2,2a,3(2*H*)-tricarboxylate (6)**

Purified by flash column chromatography on silica gel (petroleum ether/EtOAc = 15:1 (v/v)). colorless oil. (23.5 mg, 60% yield, 33.3 mg, 85% yield). **^1^H NMR** (500 MHz, CDCl_3_) δ 7.99 (d, *J* = 8.1 Hz, 1H), 7.30 (t, *J* = 7.8 Hz, 1H), 7.19 (d, *J* = 8.9 Hz, 1H), 7.01 (t, *J* = 7.5 Hz, 1H), 4.92 (s, 1H), 4.28 (s, 1H), 3.89 (s, 3H), 3.70 (s, 3H), 3.09 (s, 3H), 1.49 (s, 9H). **^13^C NMR** (126 MHz, CDCl_3_) δ 171.0, 168.4, 151.3, 145.3, 130.6, 129.8, 124.4, 123.2, 115.5, 92.1, 81.9, 74.0, 60.5, 53.0, 52.7, 50.0, 28.1. **HRMS** (ESI-TOF) Calculated for C_19_H_24_N_2_NaO_7_^+^ ([M+Na^+^]): 415.1476. found: 415.1476.

**3-(*tert*-butyl) 2-ethyl 2a-methyl (2*S*,2a*S*,7b*R*)-1-methoxy-7-phenyl-1,7b-dihydro-3*H*-azeto[3,2-*b*]indole-2,2a,3(2*H*)-tricarboxylate (7)**

Purified by flash column chromatography on silica gel (petroleum ether/EtOAc = 10:1 (v/v)). White solid. (34.7 mg, 72% yield). Melting point: >270 °C (exceeds the measurable range). **^1^H NMR** (500 MHz, CDCl_3_) δ 8.02 (d, *J* = 8.2 Hz, 1H), 7.48 (d, *J* = 7.0 Hz, 2H), 7.43 (t, *J* = 7.5 Hz, 2H), 7.37 (d, *J* = 12.1 Hz, 2H), 7.07 (d, *J* = 8.5 Hz, 1H), 4.74 (d, *J* = 7.2 Hz, 1H), 4.34 (d, *J* = 6.6 Hz, 1H), 4.22 – 4.15 (m, 1H), 4.04 (dd, *J* = 10.8, 7.2 Hz, 1H), 3.88 (s, 3H), 3.17 (s, 3H), 1.51 (s, 9H), 1.20 (t, *J* = 7.1 Hz, 3H). **^13^C NMR** (126 MHz, CDCl_3_) δ 170.9, 167.8, 151.4, 145.9, 139.8, 139.4, 130.2, 128.7, 128.5, 127.5, 123.3, 114.3, 91.2, 81.8, 73.7, 61.9, 60.1, 52.7, 49.9, 28.0, 14.1. **HRMS** (ESI-TOF) Calculated for C_26_H_30_N_2_NaO_7_^+^ ([M+Na^+^]): 505.1945. found: 505.1945.

**Copies of NMR spectra**

^1^H NMR (400 MHz, CDCl_3_) spectrum of **3aa**

^13^C NMR (100 MHz, CDCl_3_) spectrum of **3aa**

^1^H NMR (400 MHz, CDCl_3_) spectrum of **3ab**


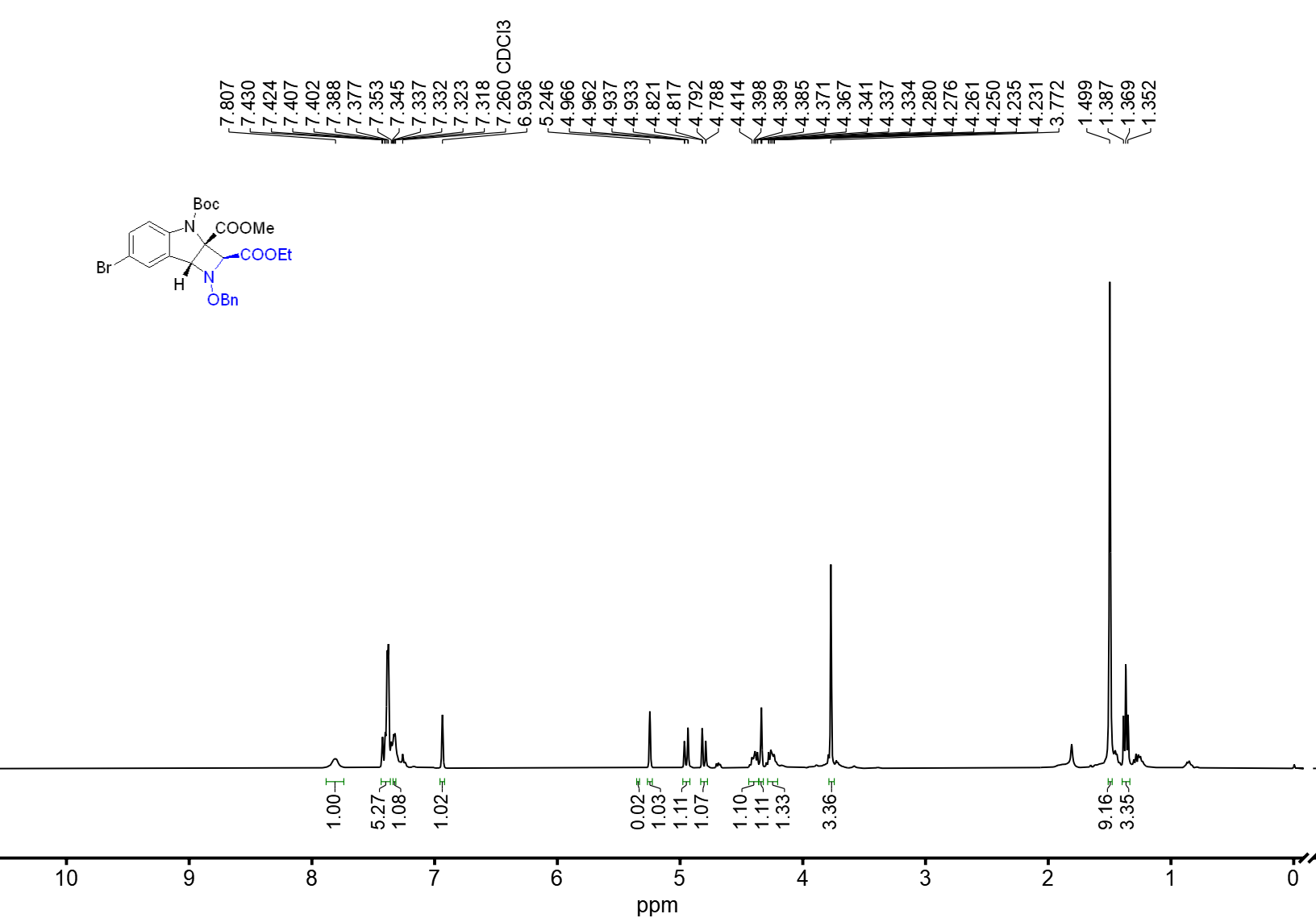


^13^C NMR (100 MHz, CDCl_3_) spectrum of **3ab**

^1^H NMR (400 MHz, CDCl_3_) spectrum of **3ac**

^13^C NMR (100 MHz, CDCl_3_) spectrum of **3ac**

^1^H NMR (400 MHz, CDCl_3_) spectrum of **3ad**

^13^C NMR (100 MHz, CDCl_3_) spectrum of **3ad**

^19^F NMR (376 MHz, CDCl_3_) spectrum of **3ad**

^1^H NMR (400 MHz, CDCl_3_) spectrum of **3ae**

^13^C NMR (100 MHz, CDCl_3_) spectrum of **3ae**

^19^F NMR (376 MHz, CDCl_3_) spectrum of **3ae**

^1^H NMR (400 MHz, CDCl_3_) spectrum of **3af**

^13^C NMR (100 MHz, CDCl_3_) spectrum of **3af**

^1^H NMR (400 MHz, CDCl_3_) spectrum of **3ag**

^13^C NMR (100 MHz, CDCl_3_) spectrum of **3ag**

^1^H NMR (400 MHz, CDCl_3_) spectrum of **3ah**

^13^C NMR (100 MHz, CDCl_3_) spectrum of **3ah**

^19^F NMR (376 MHz, CDCl_3_) spectrum of **3ah**

^1^H NMR (400 MHz, CDCl_3_) spectrum of **3ai**

^13^C NMR (100 MHz, CDCl_3_) spectrum of **3ai**

^1^H NMR (400 MHz, CDCl_3_) spectrum of **3aj**

^13^C NMR (100 MHz, CDCl_3_) spectrum of **3aj**

^1^H NMR (400 MHz, CDCl_3_) spectrum of **3ak**

^13^C NMR (100 MHz, CDCl_3_) spectrum of **3ak**

^1^H NMR (400 MHz, CDCl_3_) spectrum of **3al**

^13^C NMR (100 MHz, CDCl_3_) spectrum of **3al**

^19^F NMR (376 MHz, CDCl_3_) spectrum of **3al**

^1^H NMR (400 MHz, CDCl_3_) spectrum of **3am**

^13^C NMR (100 MHz, CDCl_3_) spectrum of **3am**

^1^H NMR (400 MHz, CDCl_3_) spectrum of **3an**

^13^C NMR (100 MHz, CDCl_3_) spectrum of **3an**

^1^H NMR (400 MHz, CDCl_3_) spectrum of **3ao**

^13^C NMR (100 MHz, CDCl_3_) spectrum of **3ao**

^1^H NMR (400 MHz, CDCl_3_) spectrum of **4aa**

^13^C NMR (100 MHz, CDCl_3_) spectrum of **4aa**

^1^H NMR (400 MHz, CDCl_3_) spectrum of **4ab**

^13^C NMR (100 MHz, CDCl_3_) spectrum of **4ab**

^19^F NMR (376 MHz, CDCl_3_) spectrum of **4ab**

^1^H NMR (400 MHz, CDCl_3_) spectrum of **4ac**

^13^C NMR (100 MHz, CDCl_3_) spectrum of **4ac**

^1^H NMR (400 MHz, CDCl_3_) spectrum of **4ad**

^13^C NMR (100 MHz, CDCl_3_) spectrum of **4ad**

^1^H NMR (400 MHz, CDCl_3_) spectrum of **4ae**

^13^C NMR (100 MHz, CDCl_3_) spectrum of **4ae**

^1^H NMR (400 MHz, CDCl_3_) spectrum of **4af**

^13^C NMR (100 MHz, CDCl_3_) spectrum of **4af**

^1^H NMR (400 MHz, CDCl_3_) spectrum of **4ag**

^13^C NMR (100 MHz, CDCl_3_) spectrum of **4ag**

^1^H NMR (400 MHz, CDCl_3_) spectrum of **5aa**

^13^C NMR (100 MHz, CDCl_3_) spectrum of **5aa**

^1^H NMR (400 MHz, CDCl_3_) spectrum of **5ab**

^13^C NMR (100 MHz, CDCl_3_) spectrum of **5ab**

^1^H NMR (400 MHz, CDCl_3_) spectrum of **5ac**

^13^C NMR (100 MHz, CDCl_3_) spectrum of **5ac**

^1^H NMR (400 MHz, CDCl_3_) spectrum of **5ad**

^13^C NMR (100 MHz, CDCl_3_) spectrum of **5ad**

^1^H NMR (400 MHz, CDCl_3_) spectrum of **5ae**

^13^C NMR (100 MHz, CDCl_3_) spectrum of **5ae**

^1^H NMR (400 MHz, CDCl_3_) spectrum of **5af**

^13^C NMR (100 MHz, CDCl_3_) spectrum of **5af**

^1^H NMR (400 MHz, CDCl_3_) spectrum of **5ag**

^13^C NMR (100 MHz, CDCl_3_) spectrum of **5ag**

^19^F NMR (376 MHz, CDCl_3_) spectrum of **5ag**

^1^H NMR (400 MHz, CDCl_3_) spectrum of **5ah**

^13^C NMR (100 MHz, CDCl_3_) spectrum of **5ah**

^19^F NMR (376 MHz, CDCl_3_) spectrum of **5ah**

^1^H NMR (400 MHz, CDCl_3_) spectrum of **5ai**

^13^C NMR (100 MHz, CDCl_3_) spectrum of **5ai**

^1^H NMR (500 MHz, CDCl_3_) spectrum of **6**

^13^C NMR (126 MHz, CDCl_3_) spectrum of **6**

^1^H NMR (500 MHz, CDCl_3_) spectrum of **7**

^13^C NMR (126 MHz, CDCl_3_) spectrum of **7**

**References**

[1] Z. Han, L. Wang, Y. Luo, X. Cui, *Org. Chem. Front.* **2025**, 12, 3177-3183.

[2] J. Liu, S. Wu, J. Yu, C. Lu, Z. Wu, X. Wu, X.-S. Xue, C. Zhu, *Angew. Chem. Int. Ed.* **2020**, 59, 8195-8202.

[3] Liu, J. Ma, T. Wang, X.-S. Xue, C. Zhu, *JACS Au* **2024**, 4, 2108-2114.

[4] M. Zhu, X. Zhang, C. Zheng, S.-L. You, *ACS Catal.* **2020**, 10, 12618-12626.

[5] R. Kleinmans, T. Pinkert, S. Dutta, T. O. Paulisch, H. Keum, C. G. Daniliuc, F. Glorius, *Nature* **2022**, 605, 477-482.
